# Low mutational load and high mutation rate variation in gut commensal bacteria

**DOI:** 10.1371/journal.pbio.3000617

**Published:** 2020-03-10

**Authors:** Ricardo S. Ramiro, Paulo Durão, Claudia Bank, Isabel Gordo

**Affiliations:** Instituto Gulbenkian de Ciência, Oeiras, Portugal; Institute of Science and Technology Austria (IST Austria), AUSTRIA

## Abstract

Bacteria generally live in species-rich communities, such as the gut microbiota. Yet little is known about bacterial evolution in natural ecosystems. Here, we followed the long-term evolution of commensal *Escherichia coli* in the mouse gut. We observe the emergence of mutation rate polymorphism, ranging from wild-type levels to 1,000-fold higher. By combining experiments, whole-genome sequencing, and in silico simulations, we identify the molecular causes and explore the evolutionary conditions allowing these hypermutators to emerge and coexist within the microbiota. The hypermutator phenotype is caused by mutations in DNA polymerase III proofreading and catalytic subunits, which increase mutation rate by approximately 1,000-fold and stabilise hypermutator fitness, respectively. Strong mutation rate variation persists for >1,000 generations, with coexistence between lineages carrying 4 to >600 mutations. The in vivo molecular evolution pattern is consistent with fitness effects of deleterious mutations *s*_*d*_ ≤ 10^−4^/generation, assuming a constant effect or exponentially distributed effects with a constant mean. Such effects are lower than typical in vitro estimates, leading to a low mutational load, an inference that is observed in in vivo and in vitro competitions. Despite large numbers of deleterious mutations, we identify multiple beneficial mutations that do not reach fixation over long periods of time. This indicates that the dynamics of beneficial mutations are not shaped by constant positive Darwinian selection but could be explained by other evolutionary mechanisms that maintain genetic diversity. Thus, microbial evolution in the gut is likely characterised by partial sweeps of beneficial mutations combined with hitchhiking of slightly deleterious mutations, which take a long time to be purged because they impose a low mutational load. The combination of these two processes could allow for the long-term maintenance of intraspecies genetic diversity, including mutation rate polymorphism. These results are consistent with the pattern of genetic polymorphism that is emerging from metagenomics studies of the human gut microbiota, suggesting that we have identified key evolutionary processes shaping the genetic composition of this community.

## Abstract 2

Weak-effect deleterious mutations and negative frequency–dependent selection, acting on beneficial mutations, shape the dynamics of molecular evolution within the mouse gut microbiota.

## Introduction

Bacteria typically live in multispecies ecosystems [1]. One of such communities is the human gut microbiota, which is key for host health and can host up to 10^13^ bacteria and hundreds of species. Over the past decade, extensive efforts have been made to characterise the factors (e.g., diet, antibiotics) that shape the species-level composition of this ecosystem (e.g., [2–4]). More recently, it has become clear that there can be abundant genetic diversity within each species colonising a given individual [5–8]. Importantly, strain level variation is key for multiple phenotypes ranging from antibiotic resistance and virulence to colonisation resistance (e.g., [9–11]). However, little is known about how different evolutionary mechanisms—such as mutation, recombination, migration, genetic drift, and natural selection—shape bacterial genetic diversity within the mammalian gut [12].

Mutation is the raw material for natural selection. The genomic mutation rate (*U*) of DNA-based microbes is typically around 0.001 per generation [13,14], suggesting that it is shaped by general evolutionary forces, independent of phylogeny or ecological niche [13,14]. Nevertheless, within-species variation of mutation rates can be observed in natural isolates of commensal and pathogenic bacteria (typically to increase mutation rate, i.e., mutator clones; e.g., [8,15–18]). Based on the known DNA repair mechanisms, mutators with 10 to >10,000-fold increases in mutation rate could, in principle, emerge and spread [19,20]. However, from the balance between the capacity to adapt (adaptability) and robustness at maintaining current fitness (adaptedness), theory predicts that mutator clones with an increase in mutation rate of 10- to 200-fold should be the most common (e.g., [21,22]; we refer to the fold-change in mutation rate of a mutator clone as its mutator strength). Indeed, when mutator emergence was observed in vitro, very strong mutators (>300-fold) were rarely detected [23–25]. However, in in vitro experiments, a bacterial lineage evolves in the absence of the multitude of biotic interactions that occur in natural ecosystems, such as the mammalian gut microbiota. In this ecosystem, biotic interactions can rapidly fluctuate [26,27] because of changes in community composition or migration of novel lineages, often caused by community perturbations such as dietary changes or antibiotic intake [2,3,28]. Under fluctuating environments, theory predicts that mutator alleles can increase in frequency, either reaching fixation or leading to mutation rate polymorphism [22,29]. Yet knowledge of within-host mutation rate variation [15–18,30] and its temporal dynamics within a diverse multispecies community [8] remains limited.

Here, we sought to gain a better understanding of the evolutionary forces shaping bacterial evolution in the mammalian gut. We followed the long-term evolution of a new commensal strain of *E*. *coli* as it invaded and established itself in the complex ecosystem of the mouse gut after a perturbation caused by antibiotic treatment. Using an experimental evolution approach, we tracked the evolution of two defined *E*. *coli* lineages in real time. During this experiment, we observed the coexistence of lineages with mutation rates ranging from wild-type levels to 1,000-fold higher. We determine the molecular causes and suggest the evolutionary conditions that allow such strong mutators to emerge and persist within a complex microbiota. At the molecular level, we show that mutations in the proofreading (α) and catalytic (ε) subunits of DNA polymerase III cause a 1,000-fold mutation rate increase and improve hypermutator fitness, respectively. At the evolutionary level, theoretical simulations suggest that the population dynamics of the mutator lineages and their pattern of molecular evolution are compatible with deleterious mutations of weak effect. Such estimates for the in vivo mutational effects are lower than current estimates from in vitro mutation accumulation (MA) experiments (0.001 to 0.03 per generation [31–35]). This indicates that the mutational load may be lower in the mouse gut than in vitro—a result that we experimentally validate. Despite the large number of deleterious mutations observed in the mutators, we can identify multiple beneficial mutations. However, no fixations were detected for specific alleles or at the functional level (gene/operon). This suggests that the trajectories of most beneficial mutations are shaped by mechanisms such as negative-frequency dependence, temporally fluctuating selection, or metabolic/spatial niche partitioning (rather than constant positive Darwinian selection). The data reveal that the evolution of commensal bacteria within the mammalian gut is consistent with both the nearly neutral theory of molecular evolution and with the theory of genetic draft [36,37]. The first poses that most spontaneous mutations are very slightly deleterious, and the second proposes that the fate of these mutations is determined by their ability to hitchhike with linked beneficial mutations, which continuously increase in frequency.

## Results and discussion

### Emergence and maintenance of mutation rate variation in a gut commensal strain

**Fig 1:**
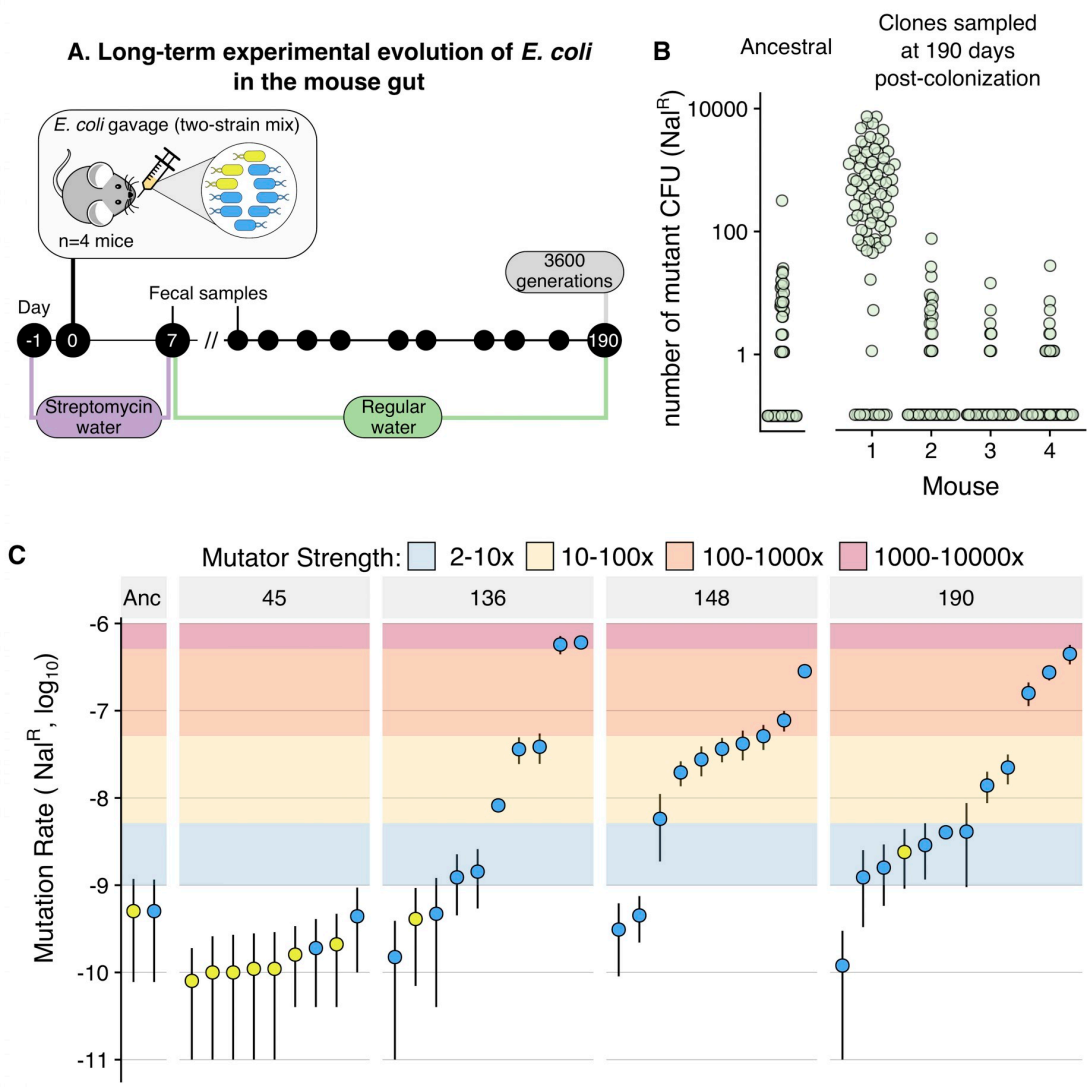
Multiple clones with variable mutation rates (up to 1,000-fold higher than ancestral) emerge and coexist during long-term adaptation to the mouse gut. (A) Scheme of the experimental design used to follow the long-term adaptation of *E*. *coli* to the mouse gut. (B) Screen for mutation rate variation shows that mutators emerged in mouse 1. A total of 83 to 90 clones, isolated from each mouse at day 190 post colonisation, were grown overnight and plated in nalidixic acid. The number of resistant colonies obtained for each clone is shown. The same procedure was carried for 96 replicates of the ancestral clones. (C) Large-scale variation for mutation rate emerges in mouse 1. Mutation rates (towards Nal^R^), measured through fluctuation tests, for multiple clones randomly isolated at different time points from mouse 1. Each point corresponds to one clone. Error bars represent 95% CIs of 10 independent growths (nonoverlapping bars indicate significant differences). Blue and yellow points correspond to clones from the CFP and YFP backgrounds. Coloured areas indicate the mutation rate fold-change of a particular evolved clone relative to the ancestral (i.e., mutator strength). CFP, cyan fluorescent protein; CFU, colony-forming units; Nal^R^, nalidixic acid resistance; YFP, yellow fluorescent protein.

To be able to study the evolution of new *E*. *coli* strains as they invade and colonise the mouse gut, we used a short-course antibiotic treatment (8 days with streptomycin), which perturbs microbiota composition and leads to a decrease in its species diversity (including killing native *E*. *coli*). With this setup, we colonised four mice with two commensal strains of *E*. *coli* (marked with two fluorescent markers: yellow fluorescent protein [YFP] and cyan fluorescent protein [CFP]; both streptomycin-resistant) and followed their dynamics for 190 days (approximately 3,600 generations, assuming 19 generations/day; Fig 1A) [38–40]. Both strains carry mutations that we previously showed to be adaptive in the mouse gut [38,39]. Mice 1 and 2 were co-colonised with a YFP-labelled strain with a 1-bp insertion in *gatC* and a CFP-labelled strain with the same *gatC* mutation and two other mutations: an IS2 insertion between *yjjP* and *yjjQ* and a 4bp insertion between *yjjY* and *yjtD*. Mice 3 and 4 were co-colonised with the same genetic backgrounds, with the single mutant expressing CFP and the triple mutant expressing YFP. All mice were co-colonised with the triple mutant at a lower starting frequency (10%), as this carries more beneficial mutations and has a higher fitness under the 7-day streptomycin treatment (S1 Fig). Throughout the entire experiment, both strains coexisted in all mice, and *E*. *coli* generally maintained a population size of >10^7^ colony-forming units (CFU)/g of faeces (S1 Fig), i.e., the typical size of its ecological niche in this host [41].

As mutation rate is a key evolutionary parameter that can evolve, both in vitro and in vivo (e.g., [8,17,23–25,30]), we screened a sample of clones from each mouse (*n* ≈ 90 per mouse) for potential changes in mutation rate (Fig 1B). In one of the four mice, we detected clones with increased mutation rate. To understand the magnitude and dynamics of the mutation rate increase in this host, we performed Luria-Delbrück fluctuation assays for several clones sampled at days 45, 136, 148, and 190 and measured their mutation rate towards nalidixic acid resistance (*n =* 10 independent growths per clone; Fig 1C). At day 45, none of the clones exhibited a mutation rate (towards resistance) significantly different from that of the ancestral (5 × 10^−10^; per cell per generation; 95% CIs for all clones overlap with those of the ancestral). However, from day 136 onwards, mutation rate varied by three orders of magnitude, ranging between 4.7 × 10^−10^ and 6 × 10^−7^/cell/generation. Similar levels of mutation rate variation were observed at days 148 and 190 (range at day 148: 3.1 × 10^−10^ to 2.8 × 10^−7^; day 190: 1.2 × 10^−10^ to 4.5 × 10^−7^ per cell per generation). This indicates that multiple mutators emerged either simultaneously or sequentially and that nonmutator clones coexisted with both strong hypermutators (mutation rates up to 1,200-fold higher than the ancestral) and with intermediate hypermutators (10- to 100-fold increases in mutation rate). Coexistence between clones differing in mutation rate by less than 70-fold has been previously observed [42–45]. However, to the best of our knowledge, our results represent the first observation of long-term coexistence (>1,000 generations, i.e., >54 days) of clones differing in mutation rate by around 1,000-fold in the gut.

These observations raised two main questions: What is the genetic basis for the observed mutation rate increase? Under what evolutionary conditions could we expect the emergence of 1,000-fold mutators and the long-term maintenance of mutation rate polymorphism?

### Mutations in the proofreading and catalytic subunits of DNA polymerase III cause hypermutability and improve mutator fitness, respectively

**Fig 2:**
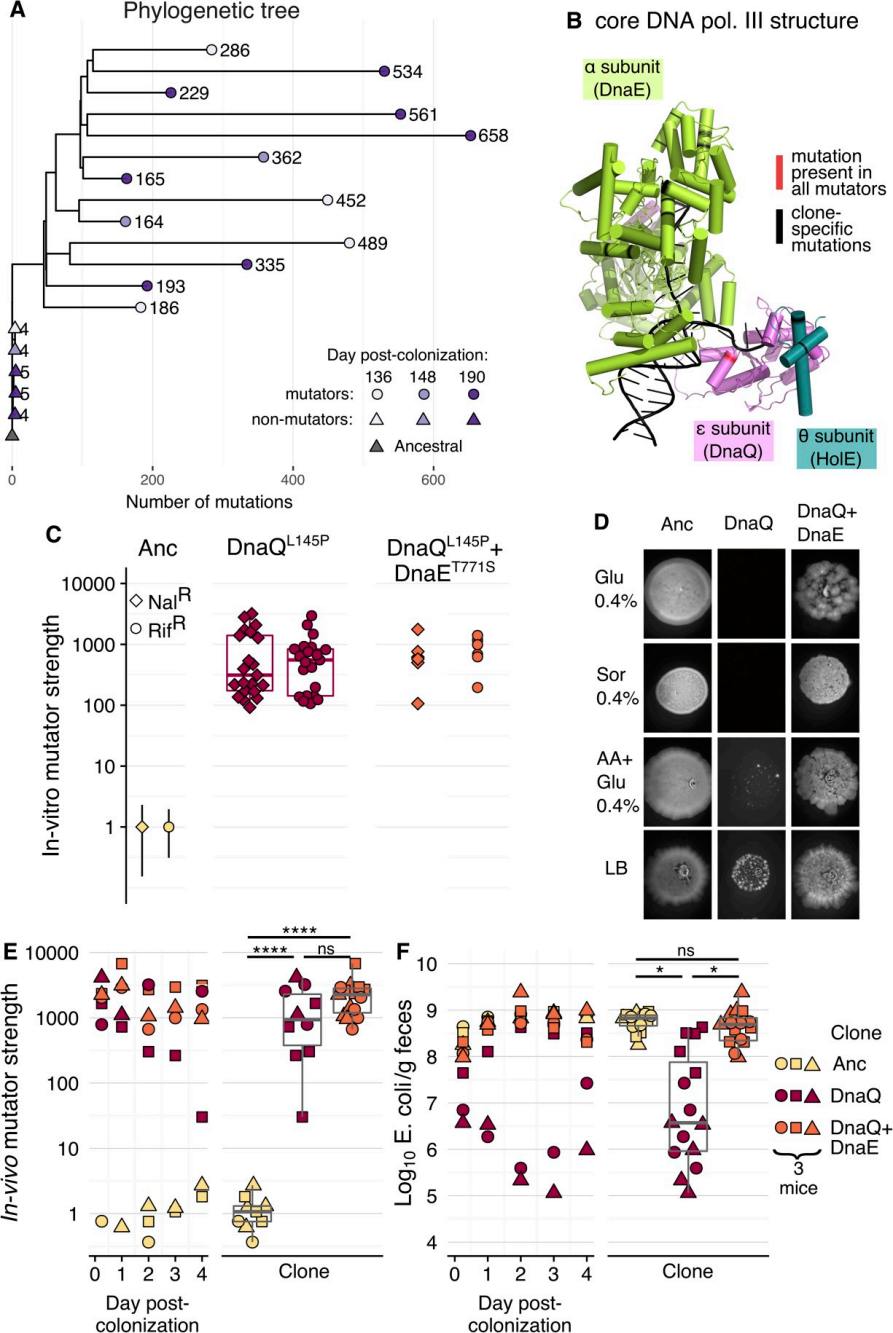
Mutations in DNA polymerase III both drive mutation rate increase by 1,000-fold (DnaQ^L145P^; ε subunit) and rescue fitness of hypermutator clones (DnaE^T771S^; α subunit). (A) Phylogenetic tree of 18 clones from mouse 1 and the ancestral strain generated from whole-genome sequence data. Mutator and nonmutators have independent evolutionary histories, with all mutators sharing 45 mutations. Branch lengths and tip labels indicate the number of accumulated mutations. Tip point colours indicate the day post colonisation at which different clones were isolated. (B) Structure of the core DNA polymerase (“pol.”) III (PDB ID: 5M1S), with each subunit in a different colour and nonsynonymous mutations highlighted in red or black. (C) In vitro fluctuation tests were performed for the ancestral clone (“Anc”), the single mutant DnaQ^L145P^, and the double mutant DnaQ^L145P^+DnaE^T771S^ (21 and 5 independent clones were tested for each mutant, respectively). Each point shows the mutation rate fold-change of a particular evolved clone relative to the ancestral (i.e., in vitro mutator strength; 10 replicates per clone for ancestral and double mutant; 5 replicates per clone for DnaQ^L145P^). For ease of visualisation, 95% CIs are only shown for the ancestral, but none of the 95% CIs of either mutant is overlapping with those of the ancestral (see S3 Fig for the mutation rate of DnaE^T771S^ and S3 Table for mutation rates and 95% CIs). (D) In vitro phenotypic growth capacity of the ancestral clone, the single mutant DnaQ^L145P^, and the double mutant DnaQ^L145P^+DnaE^T771S^ (spot assay; all inocula had the same OD_600nm_; similar results were obtained for an extended number of clones, see S4 Fig). (E) In vivo dynamics and summary box plots of the mutation frequency fold-change relative to the average mutation frequency obtained for the ancestral strain (i.e., in vivo mutator strength; similar results were obtained when measuring mutation frequency towards Nal^R^; S6 Fig); (F) In vivo dynamics and summary boxplots of *E*. *coli* CFU per gram of faeces. AA, amino acids; CFU, colony-forming units; Glu, glucose; LB, lysogeny broth; Nal^R^, nalidixic acid resistance; ns, not significant; OD_600nm_, optical density at 600 nm; PDB, Protein Data Bank; Rif^R^, rifampicin resistance; Sor, sorbitol.

To determine the cause of the observed mutation rate variation, we carried out whole-genome sequencing of 18 clones, 13 mutators and 5 nonmutators, isolated from mouse 1 (all CFP), between day 136 and 190 post colonisation (Fig 2A). Mutator and nonmutator clones accumulated 164 to 658 and 4 to 5 mutations (respectively) and followed independent evolutionary paths (Fig 2A). Observing 4 to 5 mutations in nonmutator clones after 6 months is not unexpected, given that the nonmutators are competing with mutator clones and that previous experiments showed 1 to 4 adaptive mutations in nonmutator clones after 1 month of colonisation of the mouse gut (approximately 450 generations [38,39]). All mutator clones shared 45 mutations, none of which were present in the isolates that maintained the ancestral mutation rate (Fig 2A and S1 Table). Among these, we identified a single nonsynonymous mutation in the gene *dnaQ*, which could increase mutation rate by 1,000-fold. *dnaQ* encodes the proofreading (ε) subunit of DNA polymerase III [46], the main DNA polymerase in *E*. *coli*. Following their divergence, mutators accumulated mutations in several other genes that may affect mutation rate (S2 Fig; none of these genes were mutated in the nonmutators; S2 Table). Strikingly, these included several mutations in two different subunits of DNA polymerase III: the catalytic (α) subunit (encoded by *dnaE*; mutated in 11 clones) [47] and the θ subunit (*holE*; two clones) [48]. The α, ε, and θ subunits physically interact to form the core of DNA polymerase III (Fig 2B) [20,49]. Nonsynonymous mutations in *dnaQ* were previously shown to cause mutation rate increases up to >5,000-fold [19]. Mutations in *dnaE* and to a smaller extent in *holE* were also shown to have antimutator effects when linked to *dnaQ* mutations [50,51]. This led us to hypothesise that a mutation in *dnaQ* first raised the mutation rate, which was later reduced by subsequent mutations in *dnaE*, thus leading to the observed mutation rate polymorphism. To query whether the *dnaQ* mutation caused the hypermutator phenotype, and whether *dnaE* could act as a modifier, we engineered single and double mutants with the *dnaQ* mutation (L145P; CTC → CCC) and a mutation in *dnaE* (T771S; ACG → TCG) and measured their mutation rates. We generated approximately 20 clones carrying the DnaQ^L145P^ allele, as *dnaQ* hypermutators can exhibit high mutation rate variation, possibly because of the rapid emergence of suppressors [42,51]. The *dnaQ* clones indeed showed a very high mutation rate, reaching 3,000-fold increases for both nalidixic acid and rifampicin (Fig 2C). The distribution of mutation rate estimates is bimodal, with a group of clones showing approximately 200-fold increase in mutation rate, whereas another shows an increase of approximately 1,000-fold (none of the 95% CIs overlap with the ancestral; S3 Table). Given the observed mutation rate increase of about 1,000-fold for clones isolated from mice and that it is possible for mutation rate variation to emerge during the engineering process (because of suppressors; see [51]), we suggest that the *dnaQ* mutation is responsible for the approximately 1,000-fold mutation rate increase.

Contrary to our hypothesis, the DnaE^T771S^ mutation did not cause any significant change in the mutation rate on the ancestral or the DnaQ^L145P^ genetic backgrounds (Figs 2C and S3). This suggests that the *dnaE* mutation did not act to decrease mutation rate. However, the independent occurrence of different alleles in the α subunit of DNA polymerase III (Figs 2B and S2) strongly suggests that mutations in *dnaE* could be beneficial. Consistent with this hypothesis, we observed that DnaE^T771S^ strongly improves growth of the DnaQ^L145P^ mutant (Figs 2D and S4). Remarkably, the DnaQ^L145P^ mutation is lethal in minimal media with a single carbon source (either glucose or sorbitol). Its null fitness can be rescued by complementing minimal media with amino acids or in lysogeny broth (LB), which allows the DnaQ^L145P^ mutant clones to achieve slow but visible growth (Fig 2D). Furthermore, the double mutant (DnaQ^L145P^+DnaE^T771S^) grows to levels similar to the ancestral strain across all media.

As mutation rate and spectrum can depend on the environment (e.g., [52]), we sought to determine the mutation rate of the *dnaQ* and *dnaQ*+*dnaE* mutant strains in vivo. The classical Luria-Delbrück in vitro assay, designed to avoid selection, is inappropriate for estimating the mutation rate in the gut. However, we can use Haldane’s mutation-selection balance principle to estimate the in vivo mutation rate of these clones. We colonised mice with the ancestral strain, the *dnaQ* mutant, and the double *dnaQ*+*dnaE* mutant and measured the equilibrium frequency of rifampicin-resistant (Rif^R^) clones (*n =* 3 mice for each genetic background). Under mutation-selection balance, the frequency of resistant mutants is directly proportional to the mutation rate [53]. Thus, the ratio of the Rif^R^ mutation frequency in the mutants, relative to the ancestral strain, provides an estimate of the fold-change in mutation rate (i.e., mutator strength). The mutation frequency (towards resistance) of either the single *dnaQ* mutant or the double *dnaQ*+*dnaE* mutant is about 1,000-fold higher than observed for the ancestral strain but not significantly different between mutants (Fig 2E, see also S5 Fig for comparison between mutation frequency in vivo and in an in vitro propagation; linear mixed model followed by Tukey test: clone effect: χ^2^_2_ = 31.9, *p* < 0.0001; ancestral versus *dnaQ*: *t* = −12.8, *df* = 6, *p* < 0.0001; ancestral versus *dnaQ*+*dnaE*: *t* = −14.9, *df* = 6, *p* < 0.0001; *dnaQ* versus *dnaQ+dnaE*: *t* = −1.5, *df* = 6, *p* = 0.37). Similar results were obtained when using nalidixic acid as the selection marker (S6 Fig), which targets a different region of the genome [54,55]. When comparing the niche size that each strain occupies in the mouse gut, we find that the average abundance of the *dnaQ* mutant (10^6^ per gram of faeces) is significantly lower than that of the ancestral strain (>10^8^ per gram of faeces; linear mixed model followed by Tukey test: clone effect: χ^2^_2_ = 10.2, *p* = 0.006; ancestral versus *dnaQ*: *t* = −3.7, *df* = 6, *p* = 0.022). Remarkably, the fitness defect of *dnaQ* is fully rescued by the *dnaE* mutation (Fig 2F), as the bacterial loads for the double mutant are similar to those for the ancestral strain (>10^8^ per gram of faeces; ancestral versus *dnaQ*+*dnaE*: *t* = 0.2, *df* = 6, *p* = 0.97; *dnaQ* versus *dnaQ+dnaE*: *t* = −3.5, *df* = 6, *p* = 0.029). These results are similar to those observed in vitro, when the strains grow in different nutrient sources. Taken together, our results establish that the *dnaQ* mutation massively increases mutation rate both in vitro and in vivo, albeit at a fitness cost. This cost can be recovered by a beneficial mutation in *dnaE*, without strong effects on mutation rate. The causes of mutation rate polymorphism are likely to be complex, as most mutator clones accumulated mutations in ≥3 genes that can affect mutation rates (e.g., a single clone can have mutations in *dnaQ*, *dnaE*, *recF*, *katE*, and *sodB*; S2 Fig). Interestingly, although clones with 1,000-fold increase in mutation rate (unknown genetic basis) have been observed in humans in the context of infection [17,56], to our knowledge, *dnaQ* mutators have not yet been reported in natural populations.

### Survival of hypermutators indicates that deleterious mutations are of small effect

Clonal populations with very high genomic mutation rates can quickly accumulate many deleterious mutations and experience severe impediments on fixing adaptive mutations [57,58]. Because of the accumulating mutational load, such strong mutators may continuously decay in fitness (sensu Muller’s ratchet [59,60]) in a potentially inescapable path towards extinction [61,62]. However, we observe clones with 1,000-fold increase in mutation rate that coexist with nonmutators and intermediate mutators for >1,000 generations (Fig 1C) and a high variation in the number of accumulated mutations across mutator clones (Fig 2A). Furthermore, *E*. *coli* loads in mouse 1 are approximately 10-fold lower than in any other mouse (S1 Fig), specifically after the detection of mutators, which should lead to an even faster accumulation of mutational load. These observations raise two questions: Under which conditions can such strong mutators avoid a catastrophic fitness decline, caused by the accumulation of deleterious mutations [59,60]? And what conditions allow for coexistence between mutator and nonmutator clones?

**Fig 3:**
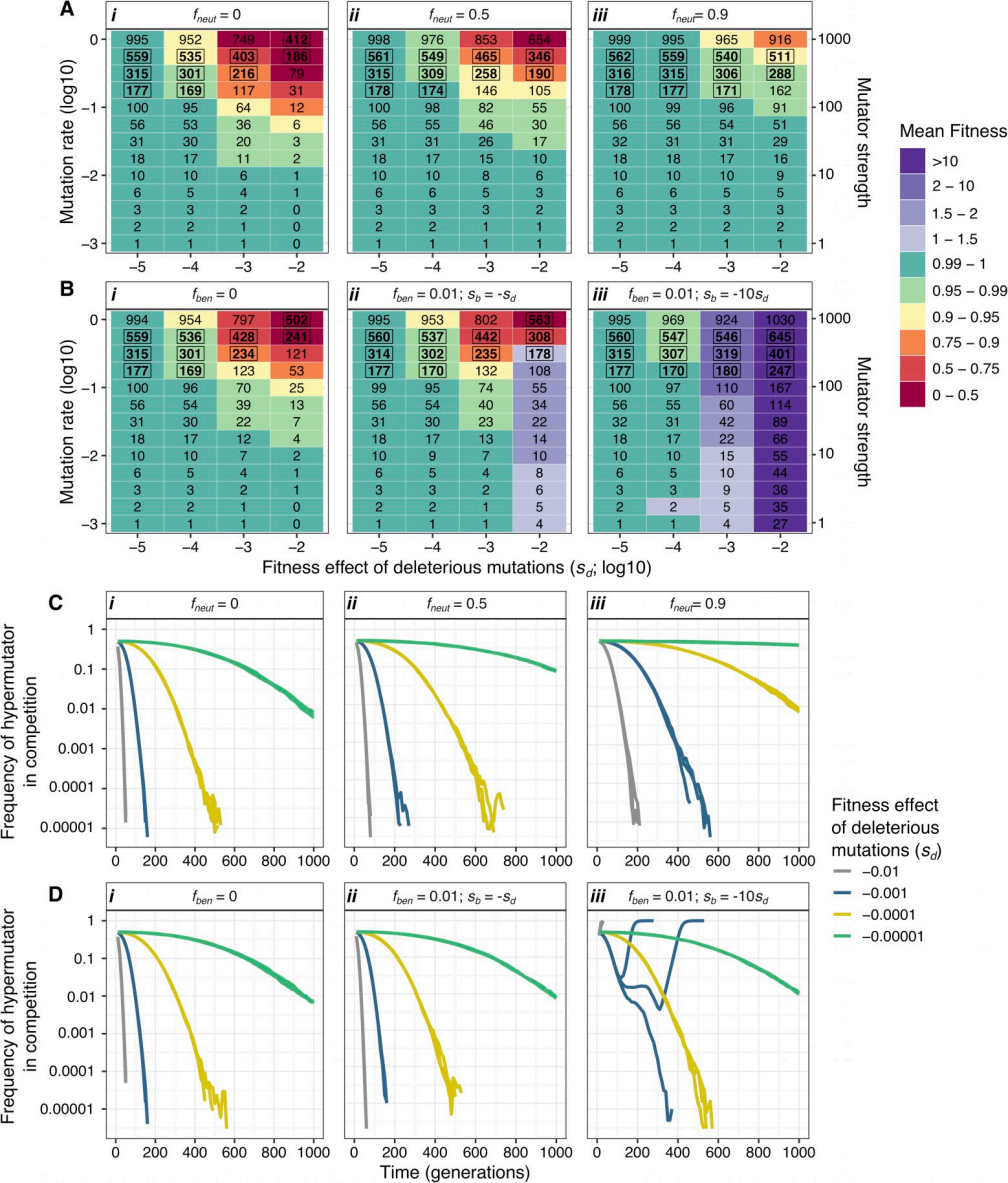
Strong, intermediate, and weak mutators can maintain high fitness and coexist if deleterious mutations have very weak effects. (A-B) Simulations with a monoclonal population. Numbers indicate the mean number of mutations, and the colour gradient indicates mean fitness after 1,000 generations (initial fitness is 1). Numbers in bold and inside a box indicate the parameter space for which there is a fit between the observed and the simulated number of mutations (*n =* 10 simulations for panel Ai and *n =* 3 simulations for all others; see S4 and S5 Tables for the mean and standard deviation of fitness and the number of mutations). (C-D) Temporal dynamics of competition between two clones differing in mutation rate by 1,000-fold (*U* = 1 and *U* = 0.001 per genome per generation; equivalent to the DnaQ^L145P^ mutant and the ancestral, respectively; dynamics of 3 simulations per parameter combination are shown). All populations had 10^6^ individuals, which evolved under different distributions of the fitness effect of mutations. (A and C) Mutations were either all deleterious with a fixed fitness effect (panel i) or neutral, with a fraction of neutral mutations (*f*_*neut*_) of 50% or 90% (panels ii-iii). (B and D) Either mutations were all deleterious (panel i), or a 1% fraction (*f*_*ben*_) was beneficial. The fitness effects of deleterious and beneficial mutations were drawn from two independent exponential distributions, with mean effects being indicated in each panel (see S7, S8, S9, and S10 Figs for a broader exploration of the parameter space and S11, S12, S13, S14, and S15 Figs for *N =* 10^5^).

To explore theoretical scenarios under which the above observations could be expected, we simulated clonal populations evolving under many mutation rate values and considered different assumptions for the fitness effects of mutations. We started by simulating the simplest model, assuming that all mutations are deleterious with a fixed and constant effect and ignoring neutral, lethal, compensatory, or beneficial mutations. We simulated populations of 10^6^ individuals (similar to *E*. *coli*’s population size in the gut; S1 Fig) under many combinations of mutation rate (*U*; per genome per generation) and the fitness effects of deleterious mutations (*s*_*d*_; per generation). We then quantified mean fitness and the number of accumulated mutations after a time similar to that over which mutator maintenance was observed (1,000 and 2,000 generations). First, we consider fixed *s*_*d*_ values and show that for clones with a wild-type mutation rate (*U* = 0.001 per genome per generation), fitness remains at approximately 1 independently of *s*_*d*_ (Figs 3Ai and S7). However, when *U* > 0.1, fitness depends on *s*_*d*_. At *s*_*d*_ values between 0.001 and 0.01, typical estimates from in vitro MA experiments with bacteria (which assume a constant *s*_*d*_ or estimate an average *s*_*d*_) [31–35], the mean fitness of lineages with *U* > 0.5 declines to <0.68 within 1,000 generations (Fig 3Ai) and is even lower after 2,000 generations (see S7 Fig and S4 Table for a broader parameter space at 1,000 and 2,000 generations). Therefore the long-term persistence of such hypermutators is unlikely. In contrast, mean fitness remains high when *s*_*d*_ ≤ 0.0001, across mutation rates spanning three orders of magnitude, and the level of variation for the number of accumulated mutations in mutator populations is similar to what we observe in the evolution experiment (indicated by the boxed numbers in Fig 3Ai). Next, we assumed a fraction *f*_*neut*_ of neutral and 1 − *f*_*neut*_ of deleterious mutations with a fixed effect (Fig 3Aii and 3Aiii), since many mutations are expected to be neutral. Neutral mutations allow hypermutators to maintain high fitness across a wider range of *s*_*d*_ values. With *f*_*neut*_ = 0.9 (Fig 3Aiii), populations with high *U* may avoid extinction, for thousands of generations, even under *s*_*d*_ = 0.01. However, irrespectively of *f*_*neut*_, when *s*_*d*_ ≤ 0.0001 mutator populations can maintain fitness at approximately 1 despite accumulating hundreds of mutations (Figs 3A and S7). These results suggest that coexistence between nonmutators and strong hypermutators is possible when *s*_*d*_ ≤ 0.0001 regardless of *f*_*neut*_, or *f*_*neut*_ = 0.9, irrespectively of *s*_*d*_. Simulations of populations with a 50:50 mixture of mutators (1,000-fold increase in mutation rate) and nonmutators competing for 1,000 generations (the minimum time over which mutators and nonmutators coexisted; Fig 1C) predict coexistence between mutator and nonmutator clones when *s*_*d*_ ≤ 0.0001 (Fig 3C) but not when *s*_*d*_ is 0.001 or 0.01. We note that lethal mutations are ignored in these simulations. These can be an important source of mutational load that could impair mutator persistence. Under a more realistic scenario, spontaneous mutations were assumed to follow an exponential distribution of deleterious effects (with mean effect *s*_*d*_ per generation). Under such conditions (Fig 3Bi and 3Di), we obtain results similar to those when *s*_*d*_ has a fixed and constant effect (Fig 3Ai and 3Ci).

Next, we evaluated the impact of beneficial mutations. In vitro estimates suggest a fraction of beneficial mutations at 1% in *E*. *coli* (*U*_*b*_ = 10^−5^ per genome per generation) with average effect *s*_*b*_ = 0.01 (per generation; [63]). Moreover, strong-effect beneficial mutations have been inferred in the mouse gut (*s*_*b*_ between 0.02 and 0.12 per generation; [39,64]). Thus, we assumed different proportions of beneficial mutations (1% or 10%), with the fitness effects of deleterious and beneficial mutations drawn from two independent exponential distributions (with mean effect *s*_*d*_ or *s*_*b*_ per generation, respectively), which may or may not be symmetrical (we tested the following mean *s*_*b*_: −*s*_*d*_, −10*s*_*d*_, 0.01, and 0.05). The occurrence of strong-effect beneficial mutations (*s*_*b*_ ≥ 0.01) leads to high variation in mean fitness under different *U* values, for large *s*_*d*_ (Figs 3Bii and 3Biii and S9 and S5 Table). Consistent with these results, coexistence between clones differing in mutation rate by 1,000-fold is short lived (<500 generations) across all *s*_*d*_ values when *s*_*b*_ ≥ 0.01 (Figs 3Dii and 3Diii and S10). Conversely, the number of observed mutations (Fig 2A) and the coexistence of clones with mutation rates differing by three orders of magnitude (for 1,000 generations) can be expected when *s*_*b*_ < 0.0001, but only if *s*_*d*_ ≤ 0.00001 (Figs 3Dii and 3Diii and S10; similar results were obtained with 10% of beneficial mutations).

The theoretical expectations, constrained by the experimental observations (in vivo accumulation of mutations and long-term maintenance of mutation rate polymorphism; Figs 1C, 2A, and 3), are consistent with low fitness effects of deleterious mutations. The same conclusion is reached under different distributions of the fitness effects of mutations and at two reasonable population sizes (see S11, S12, S13, S14, and S15 Figs for simulations under a population size of 10^5^). We note these simulations ignore temporal variation in the distribution of fitness effects of beneficial and deleterious mutations, which could affect the inferences. Thus, within the simplifying assumptions made in the simulations, the fitness effect of deleterious mutations in the mouse gut appears to be lower than what was previously inferred in vitro in rich media for *E*. *coli* from classical MA experiments (s_d_ ≈ 0.01 to 0.03, assuming a fixed effect; [31,32]) and closer to recent single-cell estimates (s_d_ ≈ 0.003; [35]).

**Fig 4:**
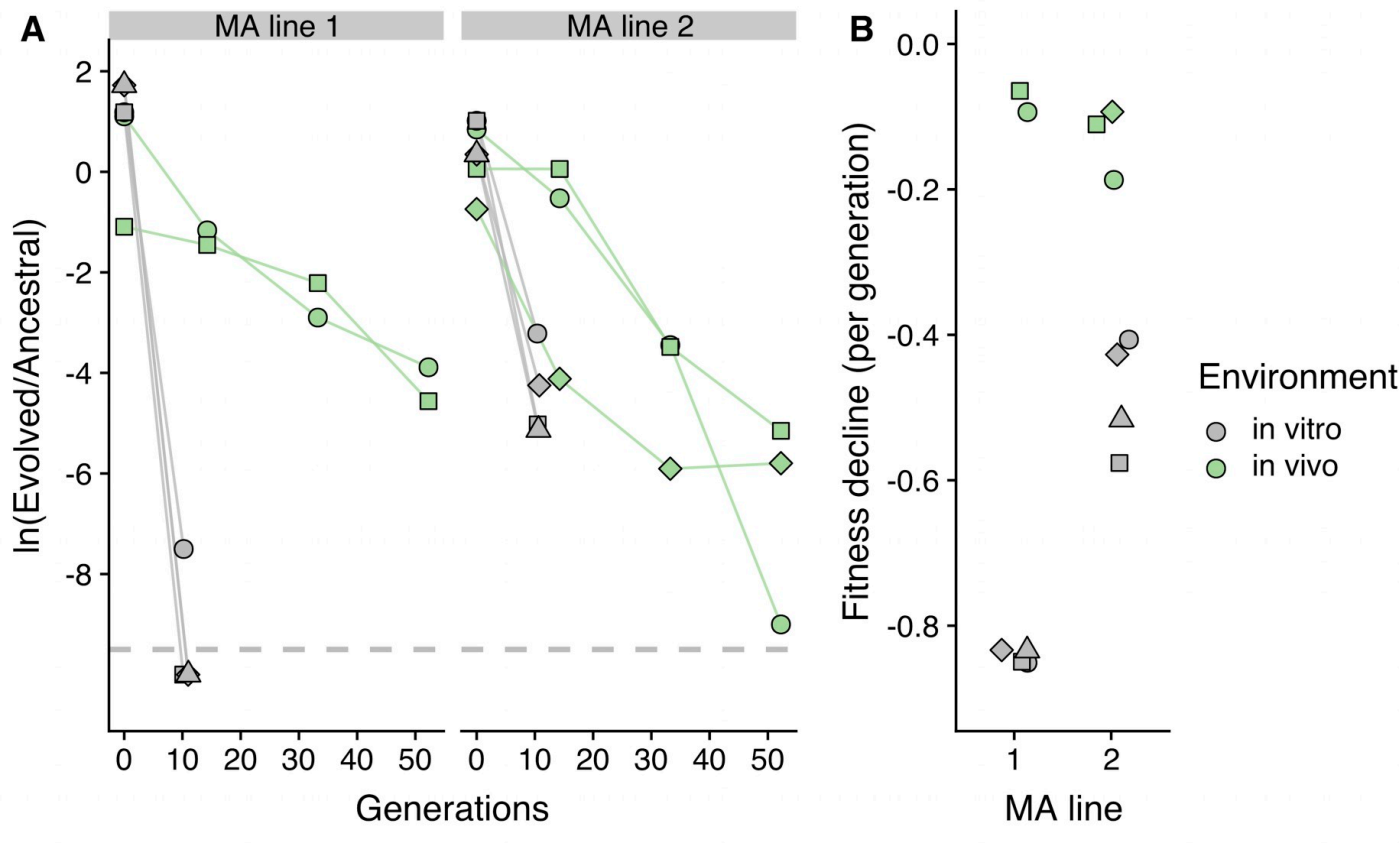
Competitive fitness assays demonstrate that the fitness effects of mutations are lower in the gut than in vitro. A short MA experiment was performed, starting from a DnaQ^L145P^+DnaE^T771S^ clone. Two lines were propagated for 4 passages (approximately 100 generations). These MA lines were propagated in conditions of nearly neutral evolution to accumulate many deleterious mutations. The evolved lines were then competed against the ancestral DnaQ^L145P^+DnaE^T771S^ clone, either in vitro (for 1 day; approximately 10 generations) or in vivo, in the mouse gut (for 3 days; approximately 50 generations). (A) Temporal dynamics of the ratio between each evolved line and the ancestral, during 50 generations of competition. The natural logarithm of the ratio of the population sizes of the two genotypes is shown. Dashed line indicates the detection limit for colonies of the evolved lines. (B) Fitness decline for the two MA lines, when competing against the ancestral strain, both in vitro and in vivo. Fitness decline is estimated as the slope of the natural logarithm of the ratio between each evolved line and the ancestral. In 3 of the 4 in vitro replicates for line 1, we could not detect any colonies of the evolved clone. To estimate a fitness decline for these replicates, we assumed 1 colony for the evolved clone. We note that the values shown in Fig 4B are the fitness declines per generation of clones that have accumulated an unknown number of mutations and thus cannot be directly compared with the estimates of the fitness effect per deleterious mutation inferred in the simulations. MA, mutation accumulation.

To complement the inference from the simulations, we sought to experimentally compare the deleterious mutational load in vivo and in vitro. We tested clones with a large number of deleterious mutations, acquired under conditions of minimal selection, for their mutational load both in vitro and in the mouse gut. We used the double mutant DnaQ^L145P^+DnaE^T771S^, which should acquire about 1 new mutation per generation, to seed a short MA experiment. Two bacterial lineages were streaked on LB agar plates and allowed to grow from a single cell to 10^8^ cells, every 24 hours (i.e., approximately 25 generations per day). After 4 passages, we isolated clones (carrying a CFP marker) from the two independent MA lines (each line should have accumulated about 100 mutations) and competed these against the ancestral DnaQ^L145P^+DnaE^T771S^ (carrying a YFP marker). As expected, fitness declined in all competitions (Fig 4A), but remarkably, the decline was much steeper in vitro than in vivo (Fig 4A and 4B). These data show that the same mutator lineage, loaded with deleterious mutations, will typically exhibit a lower mutational load in the gut than in rich LB media. Interestingly, one of the MA lines went extinct in vitro (it could not be detected in 3 of 4 replicates) but not in vivo. The combination of results indicates that levels of fitness decline are 10- and 4-fold lower for line 1 and 2 (respectively) in vivo than in vitro (linear mixed model: χ^2^_1_ = 21.8, *p* < 0.0001). This difference between in vitro and in vivo is likely an underestimate, given that natural selection is not completely absent in MA experiments [34] and because we carried the MA and the in vitro competitions in LB.

The above experimental results concur with the inference from the simulations that the fitness effect of deleterious mutations is lower in vivo than in vitro. The estimated effects of deleterious, but nonlethal, mutations are compatible with the observed dynamics of mutators in a natural environment and close to typical estimates of the fitness effect of deleterious mutations obtained from analysis of natural polymorphism (e.g., for *Drosophila*, *s*_*d*_ ≈ 10^−5^ − 10^−4^ when estimated from natural polymorphism; [65,66]). Moreover, despite the gap in knowledge regarding the causes of polymorphism in commensal bacteria [67], the high level indicates that deleterious mutations should typically have small effects [68,69]. Remarkably, by analysing polymorphism patterns in species of the human gut microbiome (including *E*. *coli*), Garud and colleagues [5] recently estimated *s*_*d*_/*μ* = 10^5^ (*μ* represents the mutation rate per site per generation). Given that our data indicate that the spontaneous mutation rate is similar in vitro and in vivo, if we take the mutation rate per site per generation estimated for *E*. *coli* from in vitro MA experiments (*μ* = 10^−10^ [70]), we obtain an *s*_*d*_ value of approximately 10^−5^, which is consistent with our results.

### Adaptive mutations target metabolism and stress resistance

Whereas weak-effect deleterious mutations can explain the observed molecular evolution patterns, they cannot explain mutator invasion. In in vitro evolution experiments, mutators have been observed to invade through hitchhiking with beneficial mutations (i.e., mutations causing the mutator phenotype increase in frequency because these occur in the same genotype as a beneficial mutation; [24,71,72]). Given the fitness defect of the *dnaQ* mutation described above (in vitro and in vivo), and because beneficial mutations can rapidly emerge during adaptation to the mouse gut [38–40,73], we propose that hitchhiking is also the most likely route driving the observed increase in mutation rate. Thus, we sought to find signatures of adaptation in the mutator clones. First, we computed the ratio of the rates of nonsynonymous to synonymous mutations, dN/dS, to detect whether there was evidence of either purifying (dN/dS < 1) or positive selection (dN/dS > 1) [74]. Consistent with the above modelling inference, in which a large number of very slightly deleterious mutations, or even neutral mutations, accumulated in the mutators, dN/dS showed a weak signal of purifying selection (dN/dS < 1 in 3/13 clones; *p* < 0.05; S16 Fig). Furthermore, no signs of positive selection could be detected using this statistic (a well-known difficulty for clonal populations).

**Fig 5:**
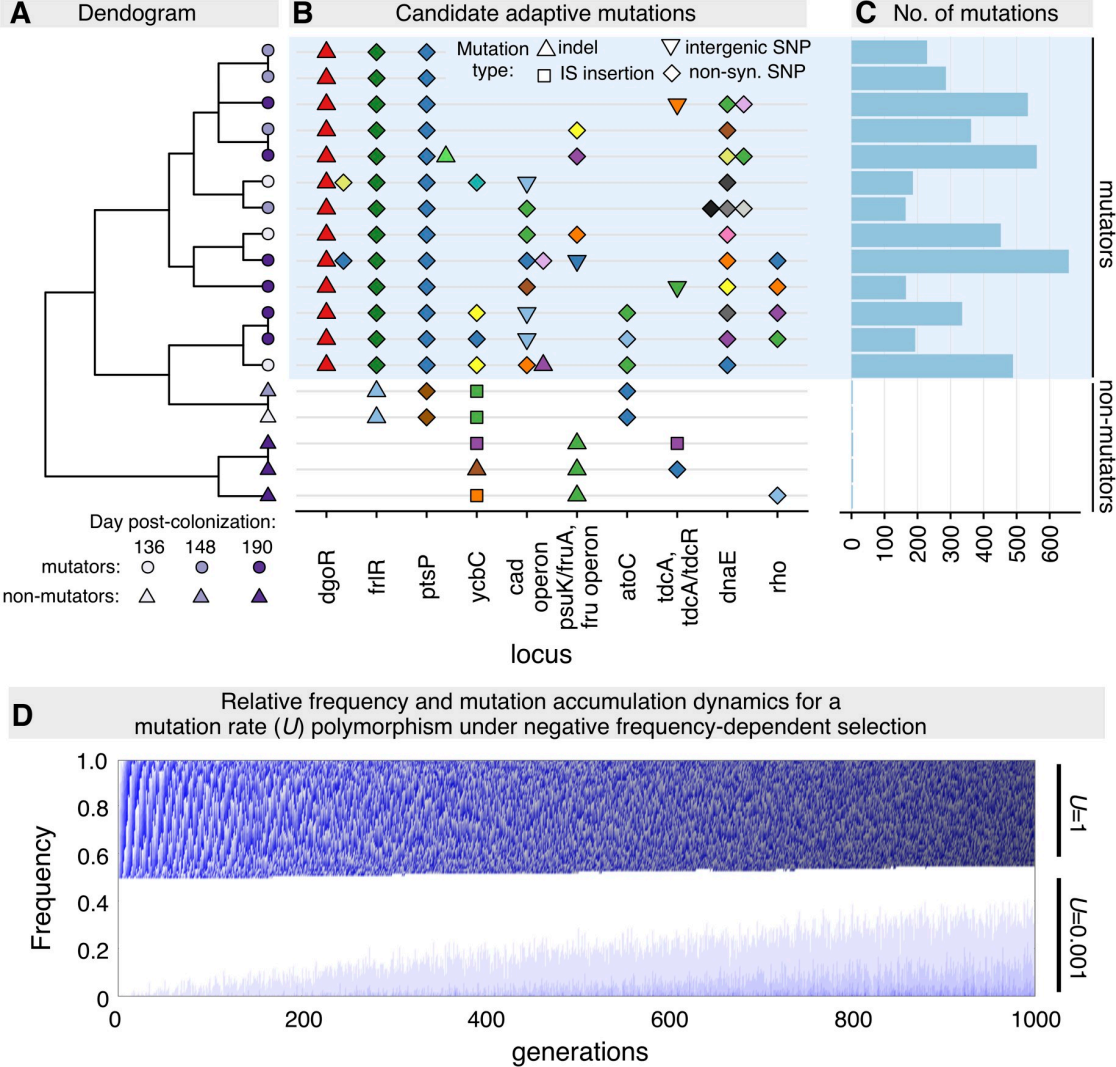
Multiple adaptive mutations accumulate in mutator and nonmutator clones without fixing, highlighting the importance of negative frequency–dependent selection. (A) Clustering of clones depending on which candidate targets of adaptation are mutated. (B) Candidate mutational targets of adaptation that are mutated in each clone (different colours indicate different alleles). All targets that showed parallelism across mice are included, plus *dnaE* and *rho*, which were identified from parallelism across mutator clones and for which we have independent evidence of adaptation. Thirty-nine additional targets were identified through parallelism across mutator clones (S6 Table). (C) Number of mutations accumulated per clone. (D) Negative frequency–dependent selection can maintain stable coexistence for 1,000 generations between two clones differing in mutation rate (*U*) by 1,000-fold. A population of 10^6^ individuals, composed by a 50:50 mixture of two clones with *U* = 1 and *U =* 0.001 (per genome per generation), was simulated for 1,000 generations, while accumulating deleterious mutations of fixed effect *s*_*d*_ = 10^−5^. The genotypes of these clones include a locus that is under negative frequency–dependent selection of strength 0.1. Darker tones of grey indicate larger numbers of mutations; shades of blue oscillate every 10 accumulated mutations. The subpopulation with *U* = 1 rapidly accumulates many deleterious mutations, reaching about 1,000 mutations by the end of the simulation. In contrast, the majority of the subpopulation with *U* = 0.001 remains without any mutations until about 400 generations and has accumulated only up to 3 mutations by generation 1,000. IS, insertion sequence; non-syn., non-synonymous; SNP, single nucleotide polymorphism.

Because of the power of our experimental design, in which the same colonising lineages evolve independently in genetically identical animals under the same diet, one can identify beneficial mutations through mutational parallelism. We isolated 3 to 4 clones per mouse (from the remaining 3 mice) 190 days post colonisation and carried out whole-genome sequencing. We then identified genes or operons that had been repeatedly mutated across hosts (i.e., parallelism; S17 Fig) as likely targets of natural selection. Using this approach, we identified 12 targets, 8 of which were also mutated in the mutator clones. Of these, *ptsP*, *frlR*, and *dgoR* were mutated in all mutators (i.e., in the mutator common ancestor), whereas 5 other targets were mutated in at least two mutator clones (Fig 5).

Most parallel targets in the mutators have a metabolic function, and some have been previously observed in other mouse gut colonisation experiments. Specifically, whereas *ptsP* is a novel target of adaptation, *frlR* and *dgoR* have been previously identified as targets of adaptation to the gut of streptomycin-treated immunocompromised or wild-type hosts, respectively [40,73]. *frlR* and *dgoR* are negative regulators of the *frl* and *dgo* operons [73,75,76], which are involved in carbon metabolism, namely of fructoselysine and galactonate (respectively). *ptsP* encodes a phosphoenolpyruvate-protein phosphotransferase, which is thought to be involved in the response to nitrogen availability and to several stresses (e.g., salt, pH) [77–79], both important pressures in the gut [80–82]. Of the three targets, *dgoR* is the most likely candidate for the adaptive mutation with which the *dnaQ* mutator allele could have hitchhiked, since *dgoR* is mutated in clones from all other mice, but only in the mutator clones from mouse 1. Furthermore, *dgoR* is the main target of adaptation when a uropathogenic *E*. *coli* colonises laboratory mice of a different genetic background [73]. This suggests that mutations in *dgoR* are strongly adaptive and such adaptation transcends the specific *E*. *coli*–mouse strain combinations. Interestingly, the mutations targeting *dgoR* and *frlR* (in this and in previous studies) often involve IS insertions, indels, or nonsense mutations. These are likely to lead to loss of function of the negative regulators and, thus, overexpression of the regulated operons. Such adaptive mutations are expected if galactonate and fructoselysine are present in the gut as limiting resources for *E*. *coli*. This form of adaptation has been previously shown to occur in the case of galactitol [64].

After divergence from their common ancestor, the mutators acquired potentially adaptive mutations in the *cad* operon, *ycbC*, *atoC*, *psuK/fruA* and *fruB*, and *tdcA/tdcR*. Of these targets, mutations in the last two have previously been observed during adaptation of *E*. *coli* to the gut of immune-competent or immunocompromised mice [40,41]. Many of the adaptive targets are functionally important for regulating carbon (*dgoR*, *frlR*, *atoC*, and, potentially, the *cad* operon) [77,83–85] or nitrogen metabolism (*ptsP* and *tdcA/tdcR*) [79,86,87], stress resistance (*ptsP*, *cad* operon, *ycbC*, and, potentially, *atoC*), and peptidoglycan biogenesis (*ycbC*) [77,83–85]. Interestingly, *ptsP*, *atoC*, and the *cad* operon appear to be involved in regulating both nutritional competence and stress resistance, which suggests that such mutations may modulate a potential trade-off between nutritional competence and stress resistance, which is well described in in vitro experiments and which is also thought to be important in vivo [81,82,88]. Together with *dgoR*, the *cad* operon is the only other mutational target that occurs in all other mice but is specific of mutator clones in mouse 1.

As the mutator clones have largely independent evolutionary histories (long terminal branches in Fig 2A), the concept of parallel evolution can also be used to identify adaptive targets that were specific to the mutator lineages. However, as the mutation rate is extremely high, parallel mutations can occur just by chance. Thus, we used simulations and conservative filters to identify genes for which the observed number of nonsynonymous single nucleotide polymorphisms (SNPs) is significantly higher than what would be expected if the observed mutations were randomly distributed (see Methods; [89]). Forty-one genes were identified with this method. Among these, we found *dnaE* and *cadC*, which had been identified as adaptive via direct experiments or through parallelism across hosts. Moreover, *rho* was also identified as a parallel target among mutators and is also mutated in one nonmutator clone, as well as during adaptation of adherent-invasive *E*. *coli* to the mouse gut [90]. These results suggest that this method is capable of identifying beneficial mutations. Of the identified genes, >50% were annotated to the following cluster of orthologous genes (COG) categories: energy production/conversion (5 genes), amino acid transport/metabolism (5 genes), signal transduction (5), and transcription (4). Among the latter two categories, most genes were involved in regulating carbon or amino acid metabolism (S6 Table). This indicates that a large fraction of mutator-specific adaptive mutations are also involved in nitrogen or carbon metabolism, which is in line with the above results.

### Partial selective sweeps structure the high polymorphism of a commensal strain evolving in the mouse gut

Among the tens of adaptive mutations that we identified, none reached fixation. Indeed, even after 190 days of evolution (approximately 3,600 generations), no complete sweep could be observed, neither of a single mutation (hard sweep) nor of multiple, functionally equivalent lineages (soft sweep). This indicates that most beneficial mutations in this ecosystem are only conditionally beneficial. Thus, a more complex form of selection is likely causing the observed polymorphism. This could be mediated by strong clonal interference or spatial or metabolic structuring of niches within the gut and/or caused by forces such as temporally fluctuating or negative frequency–dependent selection (NFDS) (in which the fitness of a genotype increases as its relative abundance decreases). Despite the simplicity of the simulations (Fig 3), these suggest that strong clonal interference is probably not a main mechanism, as simulations with *s*_*b*_ ≥ 0.01 always lead to fixation of one of the competing clones (Figs 3D and S10 and S15), although coexistence is possible when *s*_*b*_ ≤ 0.001 (and *s*_*d*_ ≤ 0.0001). However, such low *s*_*b*_ values are at odds with the observation of strong mutational parallelism across the four animals and with previous estimates of the fitness effects of beneficial mutations in the mouse gut (which ranged from 2% to 12%, with an average of 5% [38–40,64]). A stronger candidate mechanism that could lead to coexistence under a wide range of effects of deleterious mutations is NFDS, which we have previously shown to be operating in the mouse gut [39,64]. To query whether NFDS plays a role in the observed dynamics, we tested whether loss of function of *dgoR* would be under this form of selection (*dgoR* is a strong candidate for the driver mutation leading mutators to increase in frequency). When performing in vivo competitions between a *dgoR* knock-out mutant and the ancestral strain, at two different starting frequencies, we find a signature of NFDS. The *dgoR* mutant decreases in frequency when it is inoculated at a high frequency but remains at about 10% when inoculated from a low frequency (S18 Fig). As mutations in *dgoR* are highly parallel across mice (and even across different *E*. *coli*–mouse combinations), such mutations must be advantageous when rare. An important role for NFDS is also in complete agreement with three observations: lack of fixation of adaptive mutations (Figs 5 and S17), long-term maintenance of polymorphism for mutation rate (Fig 1), and maintenance of variation at a neutral locus (YFP/CFP; S1 Fig). These are not expected under a regime in which adaptive mutations are unconditionally beneficial but are consistent with a regime in which adaptive mutations are advantageous at low frequency but disadvantageous at high frequency.

To evaluate how NFDS could lead to stable coexistence between two clones with markedly different rates of molecular evolution, we simulated a population with two clones, differing in mutation rate by 1,000-fold (*U* = 1 and *U* = 0.001), whose genotypes include a locus that is under NFDS of strength 0.1. These clones further acquire recurrent deleterious mutations of effect *s*_*d*_ = 10^−5^. Under these conditions, the two clones can coexist for at least 1,000 generations at a stable relative frequency of approximately 50% (Fig 5D; coexistence was unstable in the absence of NFDS; Fig 3Ai) and with markedly different patterns of molecular evolution, similar to those observed experimentally (Fig 5C). However, we note that a signature of negative frequency–dependent dynamics may also be caused by mechanisms such as temporally fluctuating selection or different metabolic or spatial niches within the gut [26,91,92], beyond the form of NFDS that we simulated (in which fitness of a genotype depends on the relative abundance of another). For example, if there are two independent metabolic niches with different carrying capacities, the dynamics of two genotypes occupying those niches can appear to be under NFDS but may actually be determined by the absolute density of each genotype (as we have previously seen in [64]). A similar argument could be made for spatial niches, as the mammalian gut is inherently spatially structured. The relevance of these mechanisms for maintaining diversity will depend on the rate at which bacterial strains transition between such niches. Thus, further work will be needed in order to understand the relevance of different ecological and evolutionary mechanisms and how the distribution of beneficial and deleterious mutations may change along time, for determining the genetic structure of microbial populations evolving within the gut.

### Conclusions

The emergence and temporal dynamics of mutator populations can provide information on key evolutionary parameters when direct experimental measures may be difficult to obtain. Of key relevance, these can allow the fitness effects of deleterious mutations to be inferred [31–35]. Here, we studied the emergence of multiple mutator lineages during colonisation of the mouse gut, including a hypermutator with up to 1,000-fold increase in mutation rate. Under simplifying assumptions for the distribution of fitness effects of mutations, we found the pattern of molecular evolution to be consistent with effects of deleterious mutations < 10^−4^, smaller than in vitro estimates [31–35]. Competitions of MA lines, in vitro and in vivo, also indicate a lower mutational load in the mouse gut than in rich laboratory medium. The mammalian gut is a complex environment that may temporally fluctuate (e.g., variable diet or time of feeding), which could result in temporal variation of the effect of deleterious mutations, with a small time-averaged fitness effect. Indeed, we show in Fig 2D that the availability of particular resources might buffer the fitness effects of deleterious mutations. Moreover, in vitro experiments have shown that the presence of environmental stressors can lead to a reduction in the fitness effect of deleterious mutations [93]. Another mechanism for reducing the effects of deleterious mutations is revealed by the nature of the adaptive mutations. As previously observed, adaptation of *E*. *coli* to the mouse gut involved mutations linked to carbohydrate metabolism, which appear to be under NFDS, likely created by niche segregation of the different mutants [39,64]. This segregation may reduce competition between lineages and therefore buffer the effect of deleterious mutations.

The low effect of deleterious mutations should lead to weak purifying selection, which we detect in the form of a genome-wide dN/dS ≈ 1 (S16 Fig). Analysis of human metagenomics data also shows a dN/dS ≈ 1 for closely related strains with a dS ≈ 10^−5^, similar to the dS for the mutator clones studied here. However, as is typical for bacteria [94], dN/dS declines with increasing dS, reaching dN/dS of approximately 0.1 at dS ≈ 0.01, indicating strong purifying selection for divergent strains. This might suggest that different forces are affecting the genetic composition of populations on short versus long time scales. Transmission is a key force that is expected to become more important as time passes. Transmission could intensify genetic drift but also increase the strength of purifying selection. For example, deleterious mutations could be lost when transmitted across hosts if their effects are stronger in a new host (relative to the host where these emerged) or if they are hitchhiking with host-specific advantageous mutations [5,8]. More generally, understanding the effects of deleterious mutations may be of relevance for a variety of processes both within the gut microbiota (e.g., evolution of antibiotic resistance) and beyond this ecosystem (e.g., cancer evolution). As an example, it has recently been shown that tumour mutational load can be a predictor of survival for some, but not all, types of cancer [95]. Our work suggests that determining the fitness effect of deleterious mutations may be key to understand these differences.

Overall, our work suggests that the evolutionary dynamics of gut commensals may be strongly shaped by beneficial mutations, which increase in frequency but do not fix (partial sweeps), and slightly deleterious mutations that can remain in the population for long periods of time. The combination of these two effects could allow strains of the same species to coexist within the gut microbiota for extended periods with complex temporal dynamics. Novel data on temporal dynamics of genetic polymorphism in species of human gut commensals are starting to confirm this prediction, at least for some species [5–8]. As genetic diversity can impact community composition [96], more time-series data (similar to the data obtained here) are needed for a full understanding of the selective mechanisms that shape microbiota eco-evolutionary dynamics [97,98].

## Methods

### Ethics statement

This research project was ethically reviewed and approved by both the Ethics Committee and the Animal Welfare Body of the Instituto Gulbenkian de Ciência (licence references: A009.2010 and A009.2018) and by the Portuguese National Entity that regulates the use of laboratory animals (Direção Geral de Alimentação e Veterinária [DGAV], licence reference: 009676). All experiments conducted on animals followed the Portuguese (Decreto-Lei n° 113/2013) and European (Directive 2010/63/EU) legislations concerning housing, husbandry, and animal welfare.

### Bacterial strains

Strains used in this manuscript derive from the commensal *E*. *coli* K12, substrain MG1655 [99], and are named JB19-YFP, JB18-CFP, RR03-YFP, and RR04-CFP. JB19-YFP and JB18-CFP were previously described in [39]. The four strains carry a streptomycin-resistance mutation RpsL^K43R^, a deletion of the lac operon (*ΔlacIZYA*), and a fluorescent protein (YFP or CFP) linked to either a chloramphenicol (JB18 and JB19) or ampicillin (RR03 and RR04) resistance cassette that is inserted at the *galK* locus. Additionally, all strains carry mutations that were previously shown to be adaptive in the mouse gut [38,39]. JB18 and JB19 carry a 1-bp frameshift insertion in *gatC*. RR03 and RR04 carry the same 1-bp frameshift insertion in *gatC*, an IS2 insertion, and a 4-bp insertion in the intergenic regions between *yjjP* and *yjjQ* and between *yjjY* and *yjtD* (respectively). The mutations *yjjP/yjjQ*::IS2 and *yjjY/yjtD*::4 bp were acquired after adapting strain JB19-YFP to the mouse gut for 24 days [39]. P1 transductions [100] were then used to replace the construct with YFP and the chloramphenicol resistance cassette by an ampicillin resistance cassette coupled with either YFP or CFP, thus creating strains RR03-YFP and RR04-CFP.

### Mice

All experiments were carried using 6- to 8-week-old C57BL/6J male mice raised in specific pathogen-free conditions.

### In vivo experimental evolution

To simulate a perturbation of the mouse gut microbiota and allow its invasion by *E*. *coli*, we provided mice with streptomycin-treated water (5 g/L) [38,82] for 24 hours before gavage with *E*. *coli* and during 1 week post colonisation (Fig 1A). After this period, mice were given normal water until day 190 post colonisation. On the day of gavage, food and water were removed from the cage for 4 hours. Following this period, mice were gavaged with 10^8^ *E*. *coli* (suspended in 100 μl PBS). Four mice were used in this experiment, two of which were gavaged with a mixture of RR03-YFP and JB18-CFP and the other two with a mixture of RR04-CFP and JB19-YFP. Clones RR03/RR04 represented 10% of the cells in the inoculum, whereas clones JB18/JB19 represented 90%. These inocula were prepared by growing each clone separately in brain heart infusion (BHI) medium to OD_600nm_ = 2 (at 37°C on an orbital shaker). After gavage, animals were separated into individual cages, and water and food were returned to them. Colonisation of each mouse was then followed for 190 days. We estimated that 190 days of evolution in the mouse gut correspond to approximately 3,600 generations, assuming 19 generations/day, which was the generation time that we previously measured in streptomycin-treated mice [40]. In this experiment, mice are under streptomycin treatment for 1 week, which reduces microbiota diversity [28,40]. Since mice are individually caged and housing conditions impose a high level of sterility, restoration of the microbiota to a high diversity state, which would lead to increases in generation time [101], does not occur.

At each sampling point, faecal pellets were collected, weighed, and dissolved in PBS, with the remaining volume being stored at −80°C in 15% glycerol. Dilutions were made for plating in LB agar with streptomycin (100 μg/ml; or streptomycin [100 μg/ml] + ampicillin [100 μg/ml]/streptomycin [100 μg/ml] + chloramphenicol [30 μg/ml]) and plates incubated overnight at 37°C. CFP and YFP colonies were then counted on a fluorescent stereoscope (SteREO Lumar, Carl Zeiss) to estimate total numbers of *E*. *coli* and the frequency of YFP and CFP clones (S1 Fig).

### Mutator screen and fluctuation tests to estimate mutation rate

#### Mutator screen (Fig 1B)

Faecal samples isolated from each mouse at day 190 post colonisation were plated as described above. About 90 colonies per mouse were individually picked and inoculated into a different well of a 96-well plate with 200 μl of liquid LB. Plates were incubated overnight at 37°C on a plate shaker (800 rpm). After the overnight, all clones were frozen at −80°C in 15% glycerol (generating about 90 clones per mouse). Stored clones were individually defrosted into 200 μl LB in a 96-well plate and incubated overnight as described above. Ten microlitres of each culture was removed to quantify the total number of bacteria by FACS, which was approximately >10^8^ per well (only 10/430 samples were between 10^7^ and 10^8^). The remaining volume was plated in LB agar plates with nalidixic acid (40 μg/ml) to determine the number of resistant mutants that emerged during growth. Plates were incubated as described above.

#### Fluctuation tests to estimate mutation rate (Figs 1C and 2C)

Individual clones, stored at −80°C, were defrosted into PBS, plated, and grown overnight at 37°C. We then picked individual colonies to PBS and used FACS to adjust the inoculum size to 2,000 cells per replicate. Cells were then inoculated into 200 μl of liquid LB and grown in a 96-well plate for 20 to 24 hours (incubated at 37°C, 800 rpm in a plate shaker). For some clones, this was done in 1,000 μl of liquid LB with growth in 96-deep well plates. At this time point, 10 μl of each culture was removed and used to make dilutions to quantify the total number of cells by plating in LB agar plates. The remaining culture volume (and/or a 10^−1^ dilution) was then plated in LB plates with rifampicin (100 μg/ml) or nalidixic acid (40 μg/ml) to quantify the number of resistant mutants that emerged during growth. All fluctuation tests were repeated in two independent blocks. Across both blocks, 10 replicate growths per clone were carried for all clones in Fig 1C. For the results shown in Figs 2C and S3, 10 replicate growths were carried for the ancestral and the DnaE^T771S^ and DnaQ^L145P^+DnaE^T771S^ clones, whereas 5 replicate growths were carried for each DnaQ^L145P^ clones. The total number of colonies and the number of resistant colonies were then used to estimate mutation rate (and 95% CIs) with FALCOR (using the Ma-Sandri-Sarkar maximum likelihood estimator; [102–106]). This first estimates the number of mutational events (*m*), which is then divided by the total number of cells (*Nt*), leading to the estimate of mutation rate per cell per generation. Significant differences in mutation rates between clones were identified from nonoverlapping 95% CIs.

We used fluctuation tests (selecting for antibiotic resistance) to estimate mutation rates, as this allowed measuring the mutation rate for a large number of clones. We scored resistance to rifampicin or nalidixic acid, which arises through SNPs in *rpoB* or *gyrA* and *gyrB* (respectively), thus ignoring mutation rate variation along the genome [70] and neglecting insertions, deletions, and chromosomal rearrangements. The latter mutational types can contribute an important fraction of deleterious mutations, suggesting we may overestimate the deleterious mutation rate (per genome per generation; *U*_*d*_) of mutators, when we assume that mutator *U*_*d*_ increases by the same amount as *U*. We note that >93% of the mutations observed in the sequenced mutator clones are SNPs (S16 Fig), indicating that the contribution of other mutational types towards mutation rate is reduced. Moreover, it has also been estimated that single-locus fluctuation assays can overestimate mutation rates by 6-fold, relative to estimates obtained from synonymous mutations in adapting populations [107]. Thus, the rates obtained by this assay may be overestimates for both mutator and nonmutator strains. Indeed, comparisons of the mutation rate fold-change between a *ΔmutL* and a wild-type *E*. *coli* were broadly consistent between fluctuation tests and MA coupled with whole-genome sequencing [70].

### Whole-genome sequencing and analysis

#### Sequencing

Nineteen clones were sequenced from mouse 1 (where mutators emerged; 18 CFP and 1 YFP), 3 from mouse 2, 3 from mouse 3, and 4 from mouse 4. Bacterial clones were defrosted into liquid LB and grown overnight or into PBS, plated in 2–3 LB agar plates, and incubated for 24 hours at 37°C. After pelleting the cells, we extracted DNA following the procedure described in [108]. DNA library construction (with the Illumina Nextera XT kit) and sequencing were performed by the IGC genomics facility. Each clone was paired-end sequenced on an Illumina MiSeq Benchtop Sequencer. Standard procedures produced datasets of Illumina paired-end 250-bp read pairs, with mean coverage of 32-fold (19- to 45-fold per clone). The sequences (*fastq* files) can be found at the NCBI short-read archive (http://www.ncbi.nlm.nih.gov/sra; accession no. PRJNA580181). The ancestral clones had been previously sequenced for [39] (NCBI accession no. SRP063701).

#### Variant calling

*breseq* v0.26 [109], with default parameters, was used to map reads and identify mutations, using the *E*. *coli* str. K-12 substrain MG1655 genome as reference (NCBI accession: NC_000913.2). Mutations were then determined by comparison with the ancestral strains (JB18, JB19, RR03, RR04, sequenced for [39]). The reads that *breseq* failed to match to the reference genome were assembled into contigs with *SPAdes* v.3.13 (using the following parameters -m 3 -t 2--careful) [110]. All contigs with coverage >3 were then manually annotated via BLAST [111]. All annotated contigs matched the cassettes carrying the antibiotic resistance plus the fluorescent protein (with a single exception, a likely contaminant), suggesting that horizontal gene transfer did not occur during this experiment.

#### Phylogeny

We used the COMPARE function of the *breseq* gdtools suite [109,112] to create a matrix of the genetic differences between each clone. The raw mutational distances between clones were then obtained and used to construct a minimal evolution tree [113] (using the *dist*.*dna* and *fastme*.*ols* functions from the R package *ape*, v5.2; [114]), which was plotted with the *ggtree* function from the R package *ggtree* (v1.14) [115,116], generating the qualitative trees shown in Figs 2A and S16.

#### Parallel mutational targets across nonmutator clones (for Fig 5B)

These were identified by finding the mutational targets (genes and operons) that independently acquired mutations in more than one nonmutator clone (S17 Fig). As mutations are random and only 3 to 8 mutations accumulated in nonmutator clones, it is very unlikely that we would find the same gene/operon being independently mutated if such mutations did not carry a fitness benefit [117]. Using this strategy, we identified 13 mutational targets that are candidates for adaptation, 12 of which were mutated in clones isolated from different mice and a single one that acquired independent mutations in two clones isolated from the same animal.

#### Parallel mutations across mutator clones (within mouse 1; for Fig 5B)

As mutator clones have long terminal branches (Fig 2A), most of the evolutionary history of each clone is independent of the others. Thus, the mutations accumulating at these terminal branches can be used to identify parallel mutational targets. However, as the mutation rate is up to 1,000-fold higher than in nonmutator clones, there is the possibility that some mutational targets would accumulate multiple independent mutations just by chance (e.g., longer genes have a higher per-gene probability to acquire a mutation). Hence, we used a statistical approach to identify which genes accumulated more SNPs (at the terminal branches) across independent clones than would be expected by chance. We focused on SNPs, which represent the large majority of the observed mutations (S16 Fig), and on genes, as these are straightforward to define at both the sequence and functional levels. The statistical approach we used is similar to the one in [89]. Briefly, we first simulated 100 datasets in which we randomly distributed the 3,245 independent SNPs (synonymous, nonsynonymous, and intergenic) that we identified in the mutators. This randomisation process was done taking into account the observed number of each specific substitution (i.e., if it is A →T or G →C, etc.). From these simulations, we kept the nonsynonymous mutations and calculated a G score for each gene in each simulation. This involved first calculating the expected number of mutations (*E*_*i*_), as:

$$E_{i}=N_{tot}\left(L_{i}/L_{tot}\right)$$

where *N*_*tot*_ represents the total number of nonsynonymous SNPs (per simulation), *L*_*i*_ is the length of gene *i*, and *L*_*tot*_ is the total size of the coding genome. The G score for gene *i* can then be calculated as:

$$G_{i}=2N_{i}\;log_{e}\;\left(N_{i}/E_{i}\right)$$

where *N*_*i*_ is the number of nonsynonymous SNPs at gene *i*. When *N*_*i*_ = 0, we set *G*_*i*_ = 0. Using the same approach, we calculated G scores for the observed mutations at each of the mutated genes. We could then calculate a Z score for each gene *i* at which mutations were observed: $Z_{i}=\left(G_{i,obs}-\bar{G_{i,sim}}\right)/stdev\left(G_{i,sim}\right)$, where *G*_*i*, *obs*_ and *G*_*i*, *sim*_ are the G score for the observed mutations and the simulations at gene *i*, respectively (overbar and *stdev* indicate mean and standard deviation). The *Z*_*i*_ scores were then used to calculate Benjamini-Hochberg–corrected *p-*values. Furthermore, we applied two additional conservative criteria: (1) We divided the observed *N*_*i*_ and *N*_*tot*_ by 2 before estimating *G*_*i*, *obs*_. This takes into account that mutation rates can vary along the genome, in general by 2-fold [118–120]. Dividing *N*_*i*_ and *N*_*tot*_ by 2 aims to control for the possibility that significant genes are in regions of elevated mutation rate. (2) We included only genes that were mutated in at least 3 independent clones, as this leads to a similar probability of a mutation being parallel as for nonmutator clones, in which two independent mutations are considered (i.e., for nonmutator: *U*^2^ = [5 × 10^−10^]^2^ = 2.5 × 10^−19^; for mutator: *U*^3^ = [5 × 10^−7^]^3^ = 1.25 × 10^−19^). This analysis led to the identification of the parallel mutational targets described in S6 Table.

#### dN/dS estimates

As above, we used the mutations at the terminal branches to estimate dN/dS (across the entire genome) for each mutator clone, by taking the ratio of the nonsynonymous to synonymous SNPs. Given that the probability of an SNP to be nonsynonymous is not uniform across the different types of substitutions, we also estimated the expected dN/dS, given the observed mutational spectra of each clone (using a similar approach to that described in [112]). dN/dS values, shown in S16 Fig, are normalised by the expected value, and *p*-values are obtained with a binomial test.

#### DNA polymerase III structure (Fig 2B)

The DNA polymerase III structure was obtained from rcsb.org (PDB ID: 5M1S) [49,121] and edited with PyMOL to display mutations [122].

### Allelic reconstructions and P1 transductions

The mutations *DnaQ*^L145P^ (CTC→CCC) and DnaE^T771S^ (ACG→TCG) were constructed by pORTMAGE recombineering [123] in the ancestral (RR04-CFP) background, with the pORTMAGE-3 plasmid (carrying the kanamycin resistance cassette; oligomers are listed in S7 Table). The double mutant was constructed with the same method, by inserting the DnaQ^L145P^ mutation in the *dnaE* background. The presence of these mutations was confirmed by PCR and Sanger sequencing. Subsequently, we grew each clone in LB to lose the pORTMAGE plasmid (this was confirmed by streaking the clones in LB agar plates with or without kanamycin, 100 μg/ml). P1 transductions were used to construct the following clones: a YFP-expressing DnaQ^L145P^+DnaE^T771S^ mutant and a Δ*dgoR* mutant. The YFP-expressing DnaQ^L145P^+DnaE^T771S^ strain was created by replacing the CFP and ampicillin resistance cassette by a chloramphenicol resistance cassette coupled with YFP. The Δ*dgoR* strain was created by replacing the wild-type *dgoR* in RR04-CFP by the respective knock-out from the KEIO collection (strain JW5627; [124]), in which the *dgoR* sequence is replaced by a kanamycin resistance cassette.

### In vivo and in vitro mutation frequency temporal dynamics

To measure the in vivo mutation frequency (Fig 2E and 2F), we inoculated different mice with one of three different clones: ancestral (RR04), a DnaQ^L145P^ single mutant (*dnaQ*; randomly chosen from the clones with highest mutation rate), and a DnaQ^L145P^ + DnaE^T771S^ double mutant (*dnaQ+dnaE*; chosen as for single *dnaQ* clone). All clones carry the CFP marker.

Mice were provided with streptomycin-treated water (5 g/L) for 7 days before gavage with *E*. *coli*. Four hours before gavage, food and water were removed from the cages. After gavage, animals were separated into individual cages, and food and normal water (without streptomycin) were returned to them. To prepare the inoculum for these experiments, we defrosted each clone into PBS and plated in LB agar plates. After overnight incubation at 37°C, colonies were scrapped into PBS and OD_600nm_ adjusted to 2. One hundred microlitres of this suspension was then used to inoculate each mouse (i.e., 10^8^ *E*. *coli*). Independent replicate inocula were used to colonise independent mice. This procedure for preparing the inocula is different from the above because we wanted to minimise the strength of selection for compensatory mutations. These could reduce the mutation rate and/or the growth effects of the mutations (growth in liquid media, in which competition is global, is known to lead to more rapid adaptation than in solid media, in which competition is local; [125]). Faecal samples were then obtained at 6 hours and every 24 hours after gavage for 4 days and suspended in PBS. These were directly plated in LB agar plates with streptomycin and rifampicin to quantify the number of de novo Rif^R^ mutants, and dilutions were made to plate in LB agar plates with streptomycin to quantify the total number of *E*. *coli* (all clones are streptomycin-resistant). These two numbers were then used to estimate the equilibrium mutation frequency, which is proportional to mutation rate (as described in the Results section). Three mice were inoculated with each of the three clones to measure the mutation frequency towards rifampicin (Fig 2E and 2F). This experiment was repeated with two mice per clone, in order to measure the mutation frequency towards nalidixic acid (S6 Fig).

As we had estimated mutation rate in vitro with fluctuation tests, we carried a control experiment to understand if the equilibrium mutation frequency was similar between the in vitro and in vivo conditions. For this, we prepared the inocula of the three different clones as described above but started cultures with 10^6^ *E*. *coli* in 3 ml liquid LB (in 15-ml tubes). Cultures were incubated for 24 hours at 37°C in an orbital shaker. Every 24 hours, cultures were diluted 1,000-fold and allowed to grow until saturation (population size ≈ 3 to 5 × 10^9^; 10 generations per day). This procedure was repeated for 4 passages. Every day, the cells were plated as described for the in vivo experiment. The results of this experiment showed that the mutation frequency for all mutants was similar between the in vitro and the in vivo conditions (S5 Fig).

We used linear mixed models (with the R package nlme, v3.1 [126]) to analyse the temporal dynamics of mutation frequency and bacterial density, with replicate as a random effect. Both mutation frequency and bacterial density were log_10_ transformed in order to meet the assumptions made by parametric statistics.

### In vitro growth assays in different media

Bacteria were defrosted into 150 μl of liquid LB, grown overnight in a 96-well plate, and incubated at 37°C in a plate shaker (at 800 rpm). OD_600nm_ was then adjusted to 0.01, cultures were diluted 1:100, and 5 μl was spotted in agar plates and incubated at 37°C for 48 hours. After incubation, growth of each clone was imaged on a fluorescent stereoscope (SteREO Lumar, Carl Zeiss). The same inocula were tested across all media. The media used were LB and M9 minimal media with 0.4% glucose, 0.4% sorbitol, and 0.4% glucose plus the 20 proteinogenic amino acids (each at 0.05 mM) (Fig 2D).

### MA coupled with in vitro and in vivo competitions

#### MA

An MA experiment was started by streaking the DnaQ^L145P^+DnaE^T771S^ clone (expressing CFP) on two independent LB agar plates. Plates were then incubated at 37°C for 24 hours. After incubation, a single colony was picked from each plate and streaked in a new LB agar plate. This procedure was repeated for 4 consecutive passages, generating MA lines 1 and 2. After 4 passages, one colony from each independent line was grown overnight in liquid LB and stored at −80°C. We always selected the last visible colony on the streak, in order to ensure that a random colony was selected [33,34]. In each streak, colonies grow from a single cell to approximately 10^8^ cells, leading to approximately 25 generations per passage.

#### In vitro and in vivo competitions

The two MA lines (which express CFP) and the DnaQ^L145P^+DnaE^T771S^ clone (expressing YFP) were defrosted into PBS, plated in BHI plates, and incubated overnight at 37°C. After incubation, colonies were scraped into 1 ml PBS, and 100 μl of this suspension was added to 5 ml of liquid BHI (in 125-ml erlenmeyers). After 4-hour incubation, OD_600nm_ was measured and the DnaQ^L145P^+DnaE^T771S^-YFP clone was mixed with each of the MA lines, at a concentration of 10^9^ cells per ml, with a ratio of 5:1 of the MA line to the DnaQ^L145P^+DnaE^T771S^-YFP clone. This was done because preliminary experiments showed that a 1:1 ratio would lead to the MA lines being represented at <10% to 20% frequency in the inoculum.

For in vitro competitions, a 10^−5^ dilution of the suspension above was done, and 150 μl of this was then added to a 96-well plate and incubated for 24 hours (4 replicates per MA line). The inoculum and the 24-hour time point were then plated in LB agar plates with streptomycin in order to estimate the numbers of CFP and YFP colonies.

For in vivo competitions, 100 μl of the above suspension (about 10^8^ cells) was gavaged into mice (3 mice were gavaged per competition, but for line 1, only two mice became colonised). Faecal samples were then collected at 6 hours and days 1, 2, and 3 post gavage and plated in LB agar plates with streptomycin. As above, mice were provided with streptomycin-treated water (5 g/L) for 7 days before gavage with *E*. *coli*. On the day of gavage, food and water were removed from the cages for the 4-hour preceding gavage. After gavage, food and normal water (without streptomycin) were returned to all animals. All mice were single-caged for the duration of the experiment.

To determine the fitness decline (i.e., selection coefficient) of each of the MA lines, we first calculated the natural logarithm of the ratio of the evolved (MA line) against the ancestral (DnaQ^L145P^+DnaE^T771S^-YFP) at each time point. The fitness decline is then the slope of this value along time (in generations). In the in vitro experiments, the number of generations was directly estimated from the data by taking the natural logarithm of the ratio between the number of cells of the ancestral at 24 hours relative to that in the inoculum. In in vivo experiments, 19 generations per day were assumed, as has been determined in [40]. As MA line 1 went below detection level in 3 out of 4 replicates, we replaced the 0 counts by 1, in order to be able to estimate a minimum selection coefficient for these replicates.

We used linear mixed models (with the R package nlme, v3.1 [126]) to analyse the fitness decline (per generation), with MA line as a random effect.

### In vivo competitions to test for NFDS on *ΔdgoR*

Mice received streptomycin-treated water for 7 days before gavage and were gavaged as described above. Inocula for gavage were prepared by mixing the *ΔdgoR* mutant with the ancestral (RR03-YFP) at a ratio of 9:1 or 1:9.

### Statistical analysis

All analyses were performed in R, version 3.5.1 [127], using the statistical methods described in the previous sections.

### Simulations of evolving populations

Simulations were written in Mathematica [128] or C++. The annotated simulation codes are freely available online from Figshare at https://doi.org/10.6084/m9.figshare.10048427.v1.

Simulations of evolving clonal populations that can acquire deleterious mutations of fixed effects were implemented similar to those in [61]. Beginning with a mutation-free population of 10^5^ or 10^6^ individuals, at each generation mutations were drawn from a Poisson distribution with parameter *U* (mutation rate). The fitness of each individual was computed as *w*_*i*_ *= (1 − s*_*d*_*)*^*i*^, where *i* is the number of mutations carried, and *s*_*d*_ *> 0* is the deleterious selection coefficient. The next generation of the population was obtained by sampling 10^6^ individuals from the current population according to probabilities weighed by the individuals' absolute fitness. After 1,000 and 2,000 generations, respectively, we recorded the minimum number of mutations accumulated within the population (i.e., number of mutations in the least-loaded class), the mean and standard deviation of the number of mutations carried across the whole population, and the mean and standard deviation of fitness within the population.

For the model that includes neutral mutations, we performed the same simulations with two classes of mutations, a deleterious class with mutation rate U*(1 − *f*_*neut*_) and selection coefficient *s*_*d*_ and a neutral class with mutation rate U*f_*neut*_ and selection coefficient 0.

For the model with a continuous distribution of fitness effects (Figs 3B and 3D and S8, S9, S10, S12, S13, and S15), upon mutation, an individual fitness effect per mutation was drawn from an exponential distribution with mean *s*_*d*_. When including a proportion of beneficial mutations, these were drawn from an exponential distribution with mean *s*_*b*_. As described for the model with neutral mutation, the simulation was performed with two classes of mutations, and the total fitness was computed as the product of the individual fitnesses *1 + s*_*b*,*i*_ and *1 + s*_*d*,*i*_ of all beneficial and deleterious mutations an individual carried, respectively.

For any parameter combination, we considered that the number of mutations in the simulations fitted those in the experiments if the number of mutations in the simulations was contained within the range of the number of observed mutations across clones (Figs 3A and 3B and S7, S8, S9, S11, S12, and S13).

For simulations of two subpopulations with different mutation rates (Figs 3C and 3D and S10, S14, and S15), a marker locus was implemented that determines the mutation rate within the subpopulation built by its carriers. The number of individuals of subpopulation 1 in the next generation $N_{1}\left(t+1\right)$ was sampled from a binomial distribution with parameters *N* = 10^6^ and $p_{1}\left(t+1\right)=\frac{W_{1}}{\left(W_{1}+W_{2}\right)}$, where *W*_*k*_ is the total fitness of subpopulation *k* at generation *t* (i.e., the sum of all current individual fitnesses in this subpopulation). The number of individuals in subpopulation 2 was then determined as $N_{2}\left(t+1\right)=N-N_{1}\left(t+1\right)$. These simulations were performed both for fixed-effect deleterious mutations (with or without neutral mutations) and for an exponential distribution of fitness effects.

Finally, for simulations with NFDS (Fig 5D), the number of individuals of subpopulation 1 in the next generation $N_{1}\left(t+1\right)$ was sampled from a binomial distribution with parameters *N =* 10^6^ and

$$p_{1}\left(t+1\right)=\frac{\left(1+s_{freq}\left(1-p_{1}\left(t\right)\right)\right)W_{1}}{\left(\left(1+s_{freq}\left(1-p_{1}\left(t\right)\right)\right)W_{1}+\left(1+s_{freq}p_{1}\left(t\right)\right)W_{2}\right)},$$

where *s*_*freq*_ = 0.1.

## Supporting information

S1 Fig Temporal dynamics of E. coli densities and frequency of two clones over 190 days (A) and colony size variation at day 190 (B). Mouse 1 is where mutators emerged. Clones used to colonise mice 1 and 2: YFP, gatC::+C (yellow); CFP, gatC::+C, yjjP/yjjQ::IS2, yjjY/yjtD::+TTAT (blue). Clones used to colonise mice 3 and 4 have the same genotype, but the markers are swapped. Specifically, mice 3 and 4 were colonised with CFP, gatC::+C (blue); YFP, gatC::+C, yjjP/yjjQ::IS2, yjjY/yjtD::+TTAT (yellow). CFP, cyan fluorescent protein; YFP, yellow fluorescent protein.
(TIF)

S2 Fig Mutators accumulate mutations in multiple genes that can affect mutation rate. Each row represents a clone, and points with different colours represent different alleles. Genes were identified from Ecocyc (by looking at genes involved in DNA repair and replication) [129] and from [50,130–133].
(TIF)

S3 Fig In vitro mutator strengths, estimated from fluctuation tests, for the Anc, DnaQ^L145P^, DnaE^T771S^, DnaQ^L145P^+DnaE^T771S^. Data for Anc, DnaQ^L145P^, and DnaQ^L145P^+DnaE^T771S^ are the same as in Fig 2C. These are repeated here to enable visual comparison. For ease of visualisation, 95% CIs are only shown for the ancestral and DnaE^T771S^, but none of the 95% CIs of the DnaQ^L145P^ or the DnaQ^L145P^+DnaE^T771S^ mutants overlap with either the ancestral or the DnaE^T771S^ mutant (see S3 Table for mutation rates and 95% CI). Anc, ancestral clone.
(TIF)

S4 Fig In vitro growth capacity of additional clones with mutations in DnaQ^L145P^, DnaE^T771S^, and DnaQ^L145P^+DnaE^T771S^. (TIF)

S5 Fig Mutation frequency has similar dynamics in vitro and in vivo. Top: Temporal dynamics for the frequency of rifampicin-resistant mutants during an in vitro propagation in LB (left) and in vivo colonisation of the mouse gut (right) with the ancestral, *DnaQ*^L145P^ mutant, and *DnaQ*^L145P^+DnaE^T771S^ double mutant. Bottom: Box plots showing the pooled data points across all time points (colours represent different clones and shapes represent different replicates). Using a linear mixed model (with replicate as random effect), we find that there is no significant effect of either the interaction between clone and environment (i.e., in vitro or in vivo; χ^2^_2_ = 1.96, *p* = 0.38) or of experiment (χ^2^_1_ = 0.49, *p* = 0.48), with the clone being the only significant effect (χ^2^_2_ = 45.06, *p* < 0.0001). LB, lysogeny broth.
(TIF)

S6 Fig (A) In vivo dynamics and summary box plots of the mutation frequency towards nalidixic acid resistance, relative to the average mutation frequency obtained for the ancestral (i.e., in vivo mutator strength). (B) In vivo dynamics and summary box plots of E. coli CFU per gram of faeces. CFU, colony-forming units.
(TIF)

S7 Fig Simulations of a monoclonal population of 10^6^ individuals. Numbers indicate the mean number of mutations, and colour gradient indicates mean fitness after 1,000 (top) and 2,000 generations (bottom; initial fitness is 1). Mutations were either all deleterious with a fixed fitness effect (left) or neutral, with the fraction of neutral mutations (*f*_*neut*_) being 50% or 90% (middle and right panels; *n =* 10 simulations per parameter combination when *f*_*neut*_ = 0; *n =* 3 simulations per parameter combination when *f*_*neut*_ = 0.5 or *f*_*neut*_ = 0.9). See S4 Table for the mean and standard deviation of fitness and the number of mutations.
(TIF)

S8 Fig Simulations of a monoclonal population of 10^6^ individuals. Numbers indicate the mean number of mutations, and colour gradient indicates mean fitness after 1,000 (top) and 2,000 generations (bottom; initial fitness is 1). All mutations are deleterious, either with a fixed fitness effect (*s*_*d*_; left; *n =* 10 simulations per parameter combination) or with exponentially distributed effects with mean *s*_*d*_ (right; *n =* 3 simulations per parameter combination).
(TIF)

S9 Fig Simulations of a monoclonal population of 10^6^ individuals. Either mutations were all deleterious (*f*_*ben*_ = 0) or a fraction (*f*_*ben*_) of 1% (A) or 10% (B) was beneficial. The fitness effects of deleterious (*s*_*d*_) and beneficial (*s*_*b*_) mutations were drawn from two independent exponential distributions. Numbers indicate the mean number of mutations, and the colour gradient indicates mean fitness after 1,000 (top row) and 2,000 generations (bottom row; fitness starts at 1). Different *s*_*b*_ values are represented in different columns (*n =* 3 simulations per parameter combination). See S5 Table for the mean and standard deviation of fitness and the number of mutations.
(TIF)

S10 Fig Temporal dynamics of competition between two clones differing in mutation rate by 1,000-fold (*U* = 1 and *U* = 0.001 per genome per generation; equivalent to the DnaQ^L145P^ mutant and the ancestral, respectively). All populations had 10^6^ individuals, and either mutations were all deleterious (left column) or a fraction (*f*_*ben*_) of 1% (top) or 10% (bottom row) was beneficial. The fitness effects of deleterious (*s*_*d*_) and beneficial (*s*_*b*_) mutations were drawn from two independent exponential distributions.
(TIF)

S11 Fig Simulations of a monoclonal population of 10^5^ individuals. Numbers indicate the mean number of mutations, and colour gradient indicates mean fitness after 1,000 (top) and 2,000 generations (bottom; fitness starts at 1). Mutations were either all deleterious with a fixed fitness effect (left) or neutral, with the fraction of neutral mutations (*f*_*neut*_) being 50% or 90% (middle and right panels; *n =* 10 simulations per parameter combination when *f*_*neut*_ = 0; *n =* 3 simulations per parameter combination when *f*_*neut*_ = 0.5 or *f*_*neut*_ = 0.9). See S8 Table for the mean and standard deviation of fitness and the number of mutations.
(TIF)

S12 Fig Simulations of a monoclonal population of 10^5^ individuals. Numbers indicate the mean number of mutations, and colour gradient indicates mean fitness after 1,000 (top) and 2,000 generations (bottom; fitness starts at 1). All mutations are deleterious, either with a fixed fitness effect (*s*_*d*_; left; *n =* 10 simulations per parameter combination) or with exponentially distributed effects with mean *s*_*d*_ (right; *n =* 3 simulations per parameter combination).
(TIF)

S13 Fig Simulations of a monoclonal population of 10^5^ individuals. Either mutations were all deleterious (*f*_*ben*_ = 0) or a fraction (*f*_*ben*_) of 1% (A) or 10% (B) was beneficial. The fitness effects of deleterious (*s*_*d*_) and beneficial (*s*_*b*_) mutations were drawn from two independent exponential distributions. Numbers indicate the mean number of mutations, and the colour gradient indicates mean fitness after 1,000 (top row) and 2,000 generations (bottom row; fitness starts at 1). Different *s*_*b*_ values are represented in different columns (*n =* 3 simulations per parameter combination). See S9 Table for the mean and standard deviation of fitness and the number of mutations.
(TIF)

S14 Fig Temporal dynamics of competition between two clones differing in mutation rate by 1,000-fold (*U* = 1 and *U* = 0.001 per genome per generation; equivalent to the DnaQ^L145P^ mutant and the ancestral, respectively). All populations had 10^5^ individuals, and mutations were either all deleterious with a fixed fitness effect (left) or neutral, with the fraction of neutral mutations (*f*_*neut*_) being 50% or 90% (middle and right panels; *n =* 3 simulations per parameter combination).
(TIF)

S15 Fig Temporal dynamics of competition between two clones differing in mutation rate by 1,000-fold (*U* = 1 and *U* = 0.001 per genome per generation; equivalent to the DnaQ^L145P^ mutant and the ancestral, respectively). All populations had 10^5^ individuals, and either mutations were all deleterious (left column) or a fraction (*f*_*ben*_) of 1% (top) or 10% (bottom row) was beneficial. The fitness effects of deleterious (*s*_*d*_) and beneficial (*s*_*b*_) mutations were drawn from two independent exponential distributions.
(TIF)

S16 Fig Molecular phenotypes of mutator clones. (A) Phylogenetic tree for the mutator clones sequenced from mouse 1. (B) dN/dS with colours highlighting whether this is significantly different from neutral expectation. (C) Mutational spectra. Mutations accumulated at the tips were chosen for this analysis as a way to avoid counting the same mutation multiple times.
(TIF)

S17 Fig Mutations observed across nonmutator clones isolated from the four different mice (3 to 6 clones per mouse). Circles represent genomes, different coloured circles represent different mice (orange, mouse 1; blue, mouse 2; yellow, mouse 3; green, mouse 4). Grey rectangles highlight mutations that were parallel across different mice (gene labels in black) or for which multiple alleles were found within the same mouse (gene labels in grey). Small rectangles crossing a particular circle indicate mutations, with colours representing different mutation types. Note that for the figures in the main text, we always show 5 nonmutator clones from mouse 1, and there are 6 in this figure. This is because we sequenced one clone at day 190 from the YFP background. As this is not genetically related to the ancestral from which the mutators emerged, we do not show this clone in the main figures. YFP, yellow fluorescent protein.
(TIF)

S18 Fig In vivo competition between the *ΔdgoR* mutant and the ancestral, when starting from high or low frequency (*n =* 1 mouse at each starting frequency). The selective coefficient per generation (S(gen)) is indicated above each line.
(TIF)

S1 Table Mutations common to all mutator clones, i.e., present in the mutator common ancestor. Mutation in *dnaQ* is highlighted in bold, as this is the mutation responsible for the increase in mutation rate.
(XLSX)

S2 Table Mutations identified by *breseq* across all clones. (XLSX)

S3 Table Mutation rates and CIs for Figs 2C and S3. Clones in bold were used to estimate mutation frequency in vivo (both *dnaQ* and *dnaQ+dnaE*) and for the mutation accumulation (*dnaQ+dnaE*).
(XLSX)

S4 Table Simulations of a monoclonal population of 10^6^ individuals, accumulating neutral and/or deleterious mutations of fixed effect (data for Fig 3A and in S7 Fig). (XLSX)

S5 Table Simulations of a monoclonal population of 10^6^ individuals, accumulating deleterious and beneficial mutations with exponentially distributed effects (data for Figs 3B and S9). (XLSX)

S6 Table Candidate targets of adaptation, identified by parallelism across mutator clones. Genes shown pass the criteria described in the Methods. *dnaE*, *cadC*, and *rho* are shown in bold, as these were identified as adaptive through independent methods. Genes are annotated to their COG group with EggNOG v4.5.1 [134]. For the clusters Transcription and Signal transduction mechanisms, or if function is unknown or no orthologs are found, we provide a function summary from Ecocyc [129] (to enable understanding what are the functions that such genes are regulating).
(XLSX)

S7 Table List of oligomers and primers used to mutagenise and confirm mutations (through Sanger sequencing) in *dnaQ* and *dnaE*. (XLSX)

S8 Table Simulations of a monoclonal population of 10^5^ individuals, accumulating neutral and/or deleterious mutations of fixed effect (data for S11 Fig). (XLSX)

S9 Table Simulations of a monoclonal population of 10^5^ individuals, accumulating deleterious and beneficial mutations with exponentially distributed effects (data for S13 Fig). (XLSX)

## Abbreviation

CFP: cyan fluorescent protein
CFU: colony-forming units
COG: cluster of orthologous genes
IS: insertion sequence
LB: lysogeny broth
MA: mutation accumulation
NFDS: negative frequency–dependent selection
ns: not significant
Rif^R^: rifampicin-resistant
SNP: single nucleotide polymorphism
YFP: yellow fluorescent protein
