# Supplementary figures and images for: Low mutational load and high mutation rate variation in gut commensal bacteria
Source: PLoS Biol. 2020 Mar 10;18(3):e3000617. doi: 10.1371/journal.pbio.3000617 (PMC7064181; doi:10.1371/journal.pbio.3000617)

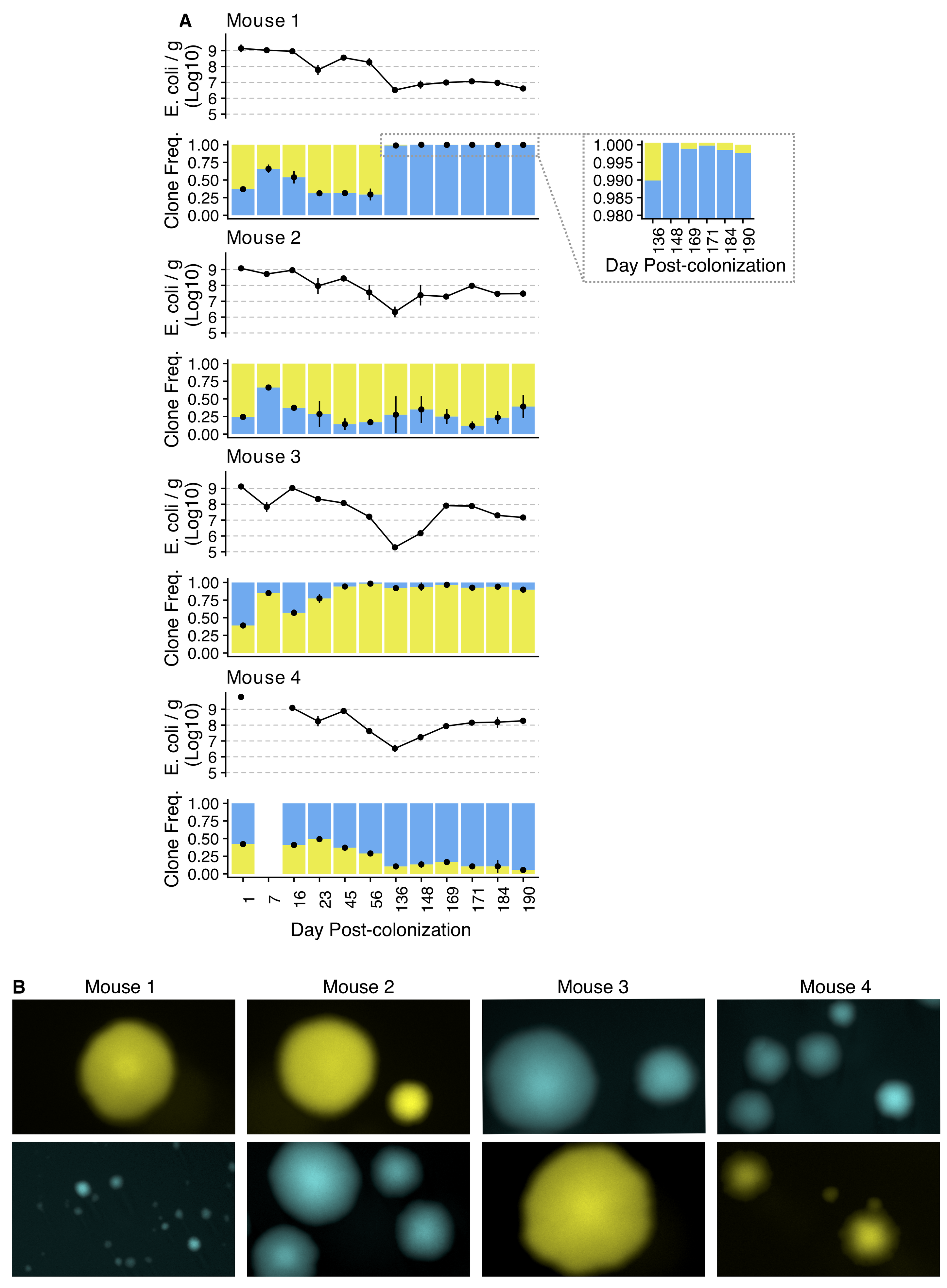

Supplement: S1 Fig — Temporal dynamics of E. coli densities and frequency of two clones over 190 days (A) and colony size variation at day 190 (B). Mouse 1 is where mutators emerged. Clones used to colonise mice 1 and 2: YFP, gatC::+C (yellow); CFP, gatC::+C, yjjP/yjjQ::IS2, yjjY/yjtD::+TTAT (blue). Clones used to colonise mice 3 and 4 have the same genotype, but the markers are swapped. Specifically, mice 3 and 4 were colonised with CFP, gatC::+C (blue); YFP, gatC::+C, yjjP/yjjQ::IS2, yjjY/yjtD::+TTAT (yellow). CFP, cyan fluorescent protein; YFP, yellow fluorescent protein. (TIF) [file pbio.3000617.s001.tif]

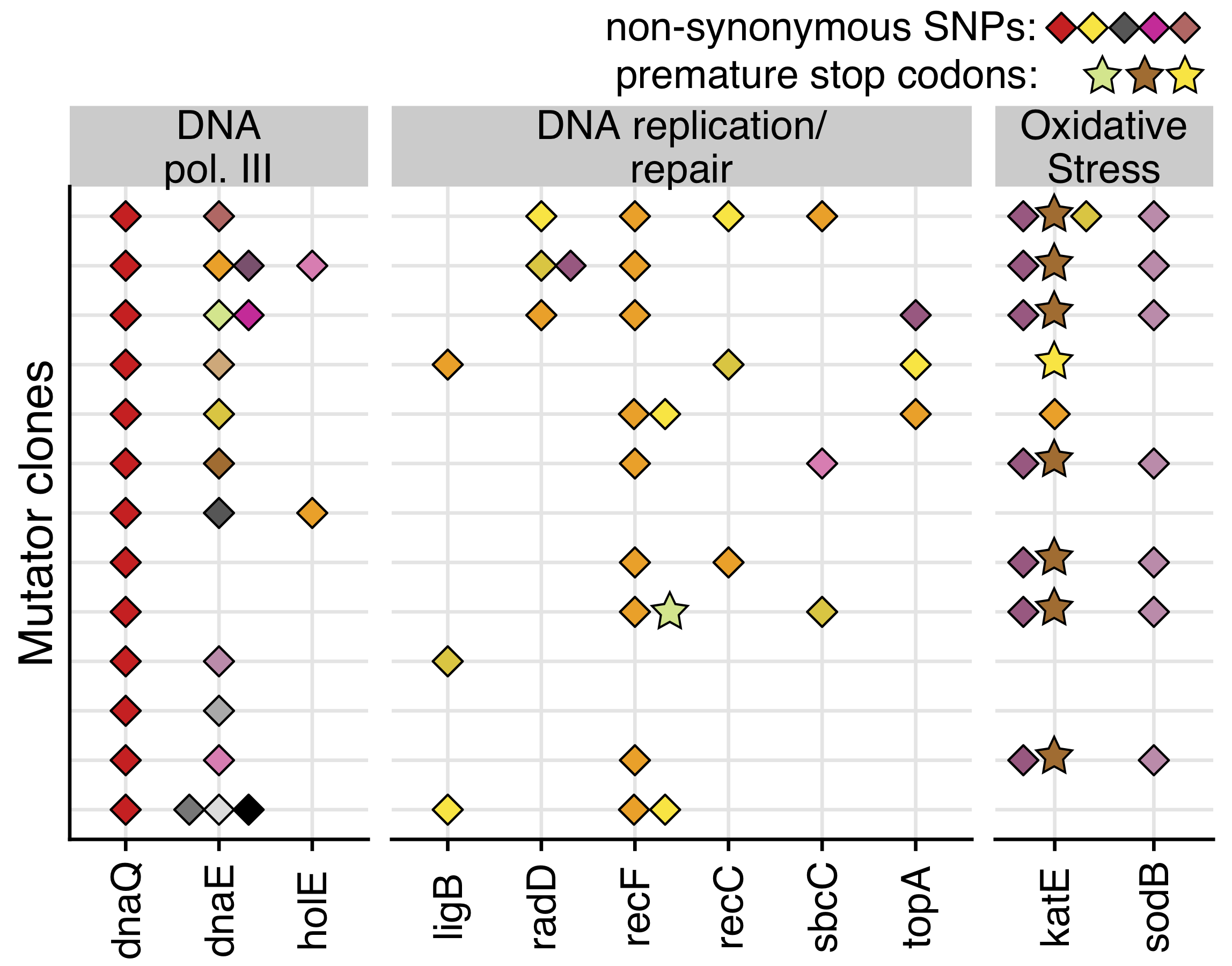

Supplement: S2 Fig — Each row represents a clone, and points with different colours represent different alleles. Genes were identified from Ecocyc (by looking at genes involved in DNA repair and replication) [129] and from [50,130–133]. (TIF) [file pbio.3000617.s002.tif]

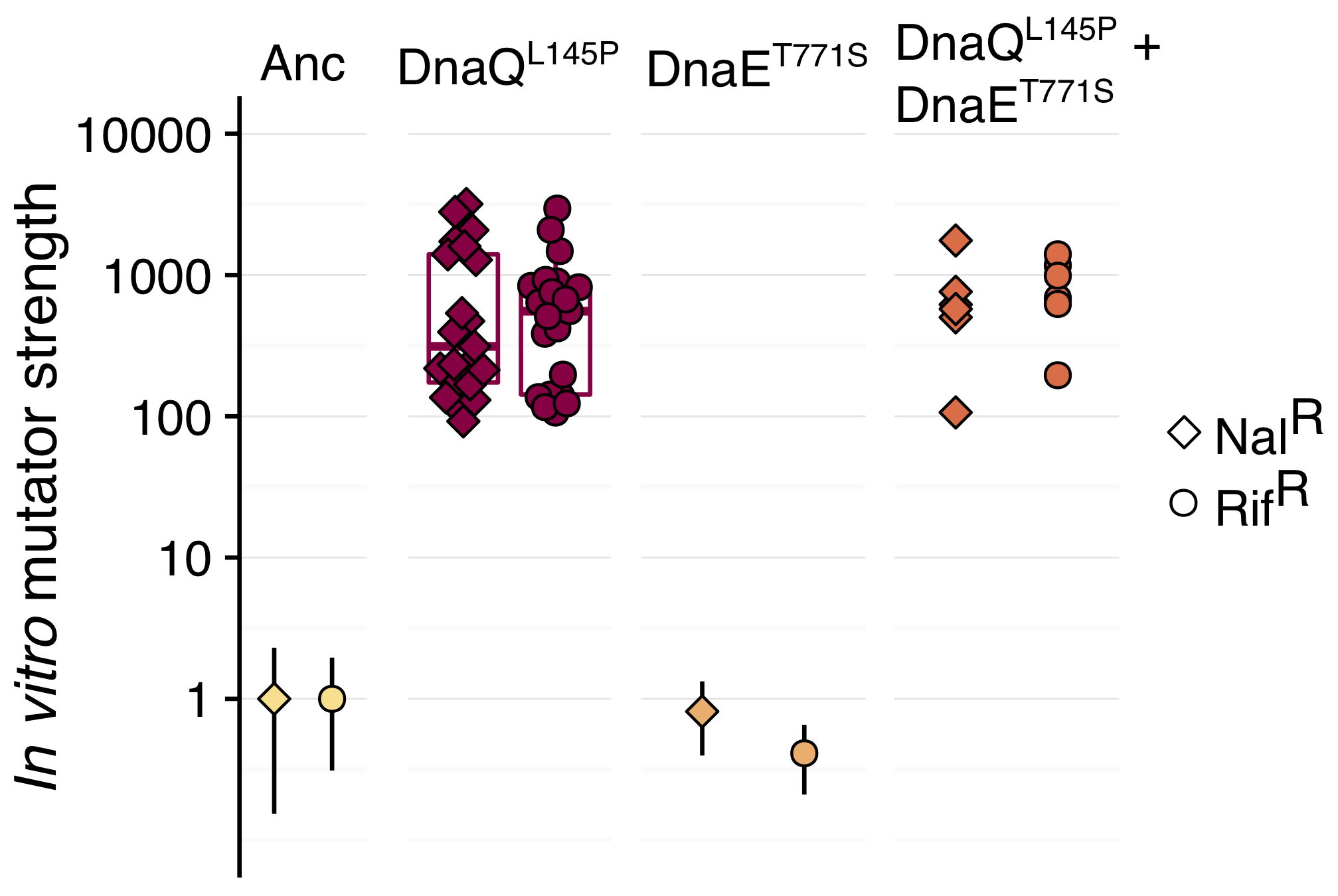

Supplement: S3 Fig — Data for Anc, DnaQL145P, and DnaQL145P+DnaET771S are the same as in Fig 2C. These are repeated here to enable visual comparison. For ease of visualisation, 95% CIs are only shown for the ancestral and DnaET771S, but none of the 95% CIs of the DnaQL145P or the DnaQL145P+DnaET771S mutants overlap with either the ancestral or the DnaET771S mutant (see S3 Table for mutation rates and 95% CI). Anc, ancestral clone. (TIF) [file pbio.3000617.s003.tif]

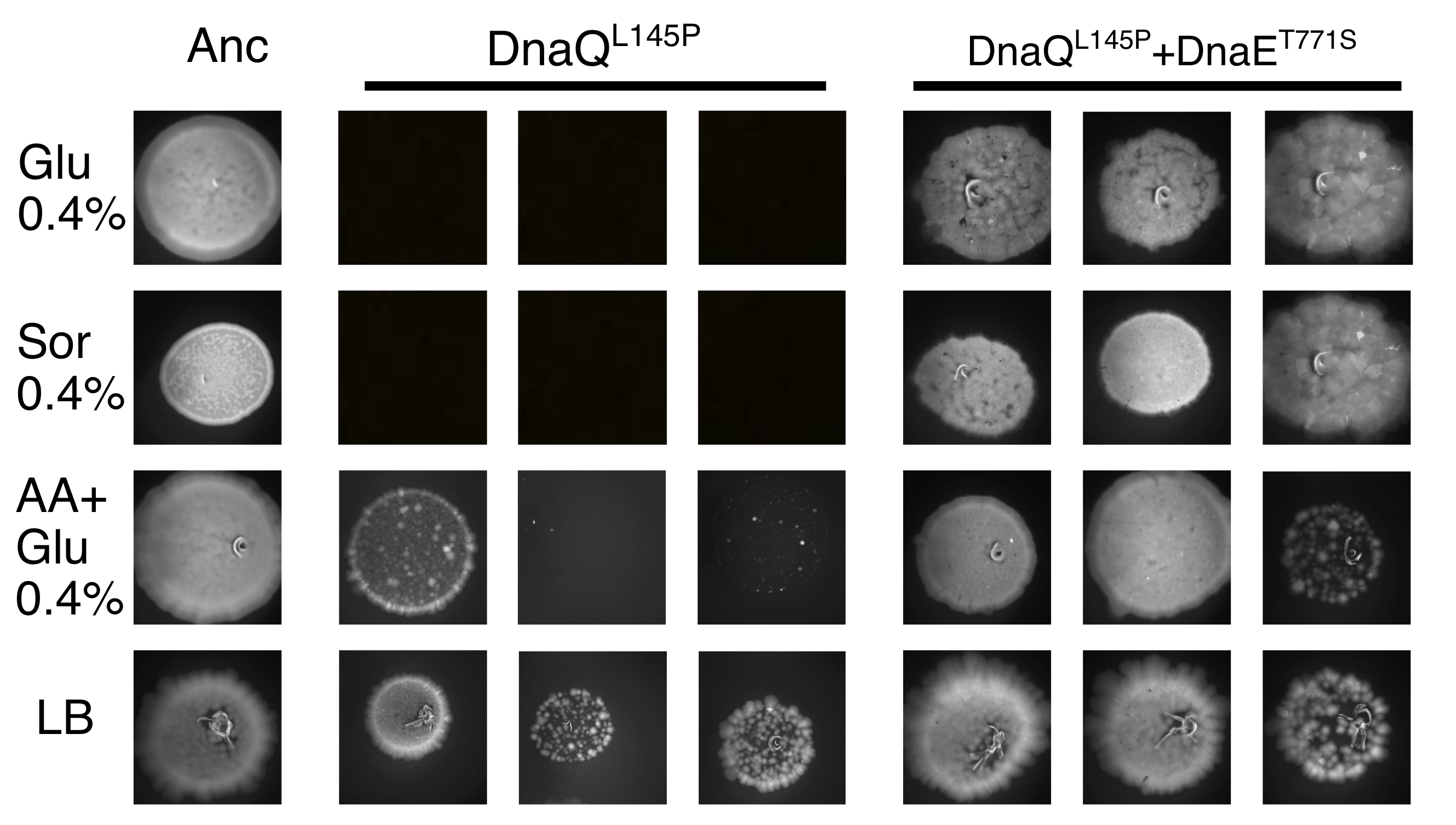

Supplement: S4 Fig — (TIF) [file pbio.3000617.s004.tif]

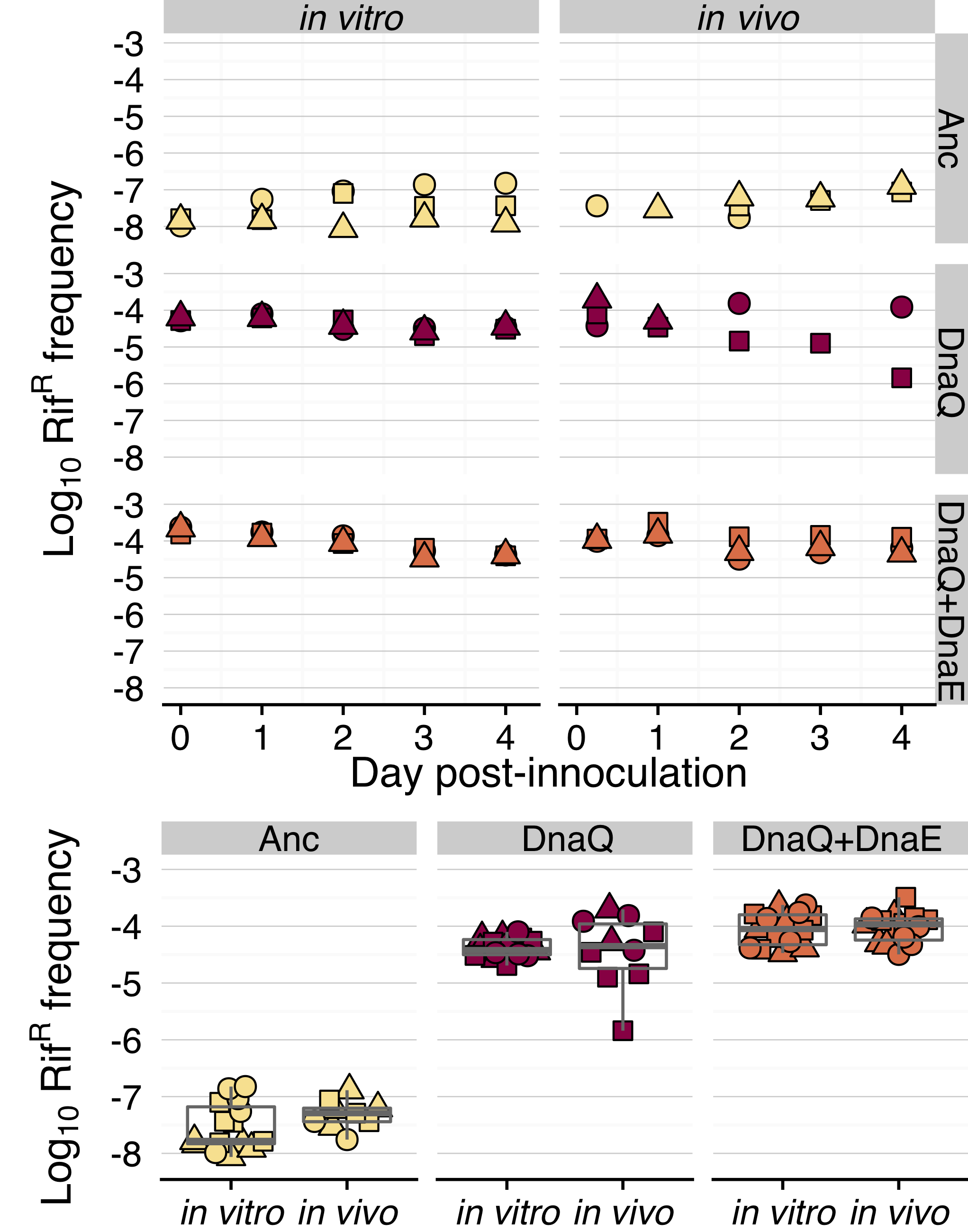

Supplement: S5 Fig — Top: Temporal dynamics for the frequency of rifampicin-resistant mutants during an in vitro propagation in LB (left) and in vivo colonisation of the mouse gut (right) with the ancestral, DnaQL145P mutant, and DnaQL145P+DnaET771S double mutant. Bottom: Box plots showing the pooled data points across all time points (colours represent different clones and shapes represent different replicates). Using a linear mixed model (with replicate as random effect), we find that there is no significant effect of either the interaction between clone and environment (i.e., in vitro or in vivo; χ22 = 1.96, p = 0.38) or of experiment (χ21 = 0.49, p = 0.48), with the clone being the only significant effect (χ22 = 45.06, p < 0.0001). LB, lysogeny broth. (TIF) [file pbio.3000617.s005.tif]

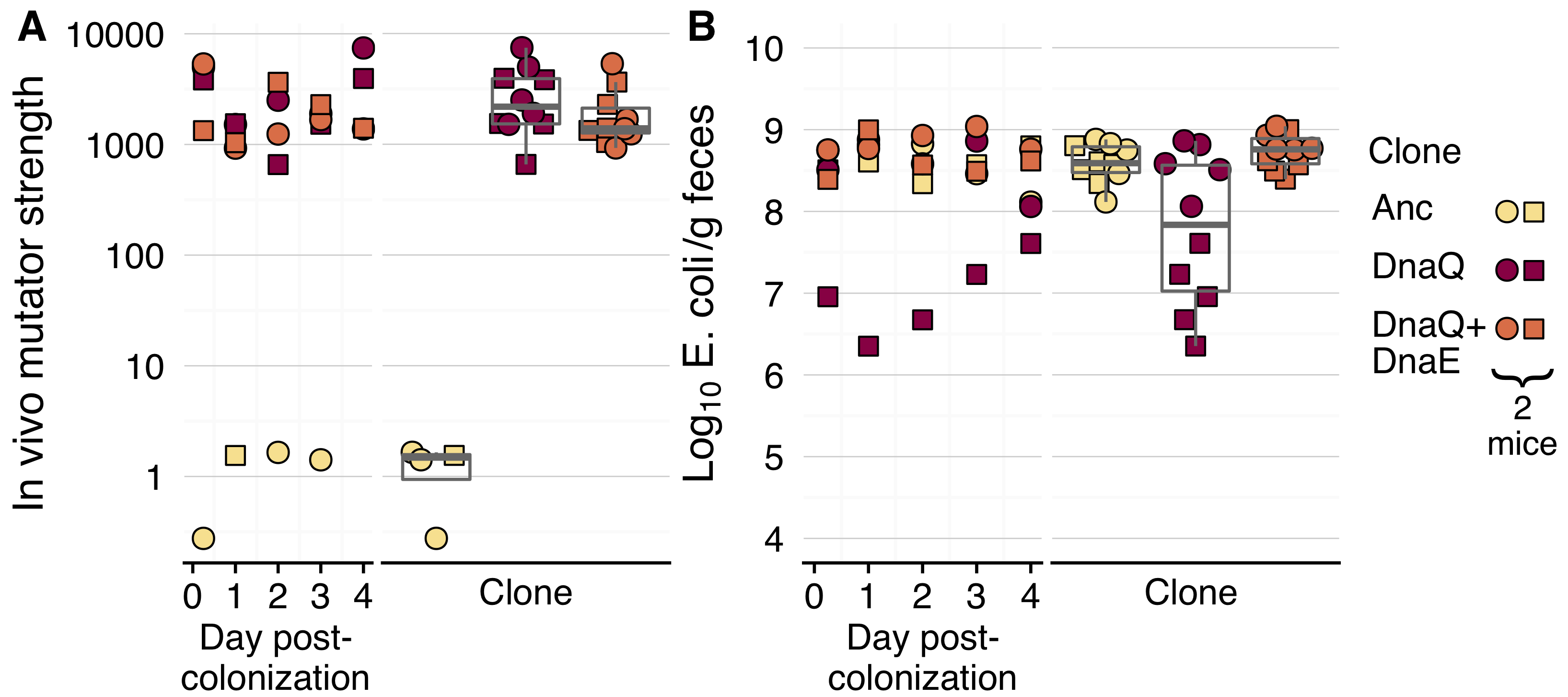

Supplement: S6 Fig — (A) In vivo dynamics and summary box plots of the mutation frequency towards nalidixic acid resistance, relative to the average mutation frequency obtained for the ancestral (i.e., in vivo mutator strength). (B) In vivo dynamics and summary box plots of E. coli CFU per gram of faeces. CFU, colony-forming units. (TIF) [file pbio.3000617.s006.tif]

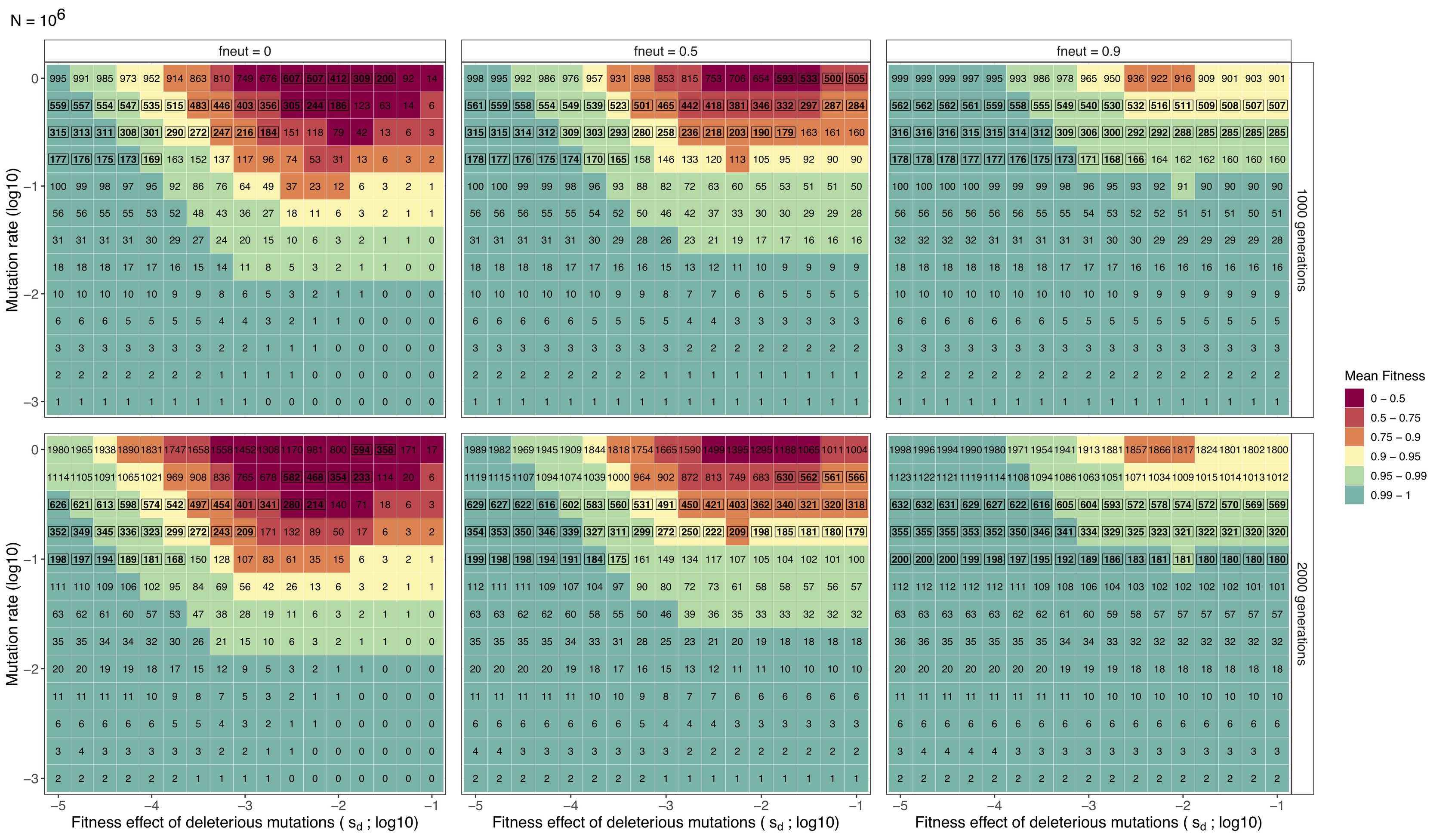

Supplement: S7 Fig — Numbers indicate the mean number of mutations, and colour gradient indicates mean fitness after 1,000 (top) and 2,000 generations (bottom; initial fitness is 1). Mutations were either all deleterious with a fixed fitness effect (left) or neutral, with the fraction of neutral mutations (fneut) being 50% or 90% (middle and right panels; n = 10 simulations per parameter combination when fneut = 0; n = 3 simulations per parameter combination when fneut = 0.5 or fneut = 0.9). See S4 Table for the mean and standard deviation of fitness and the number of mutations. (TIF) [file pbio.3000617.s007.tif]

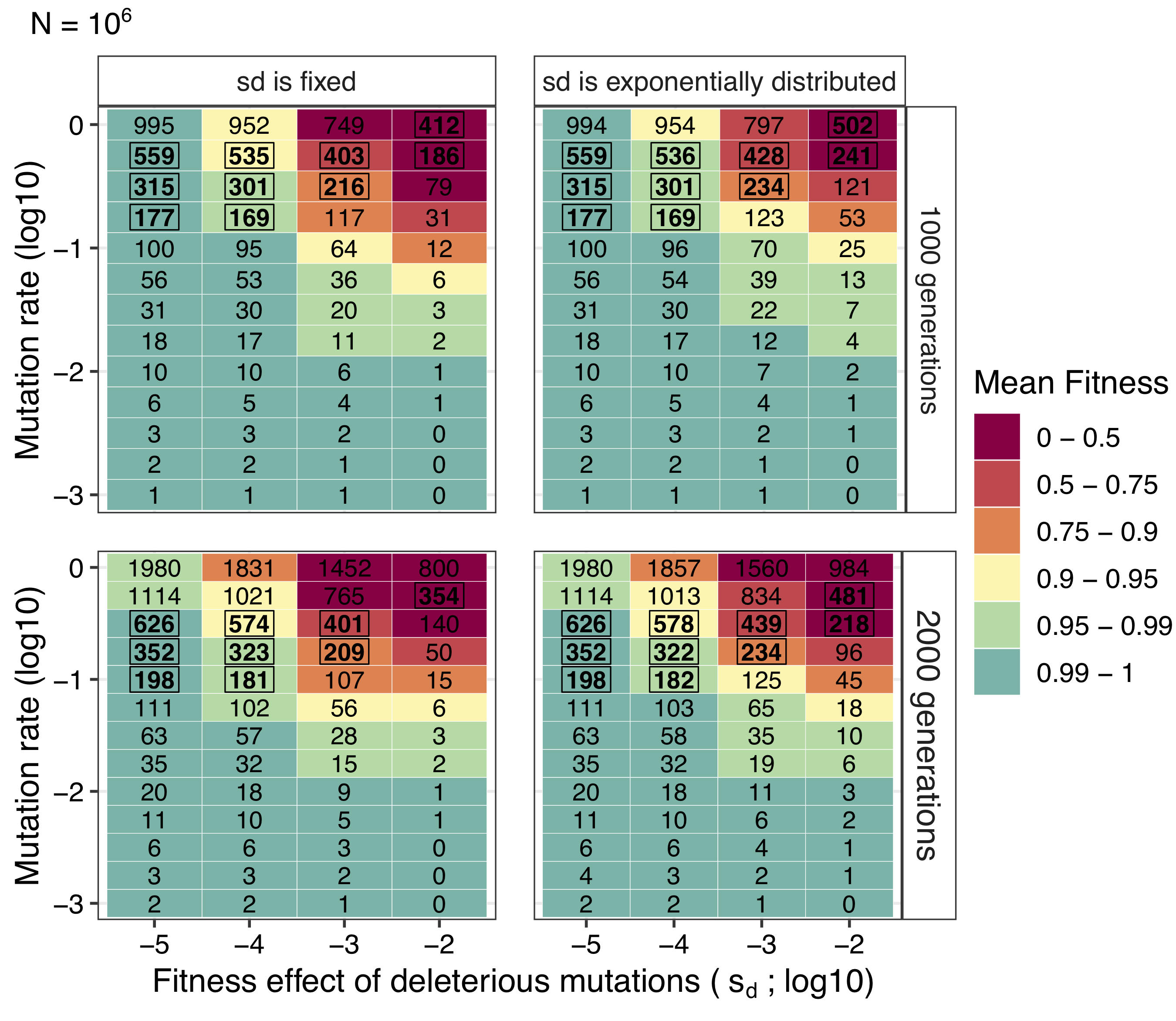

Supplement: S8 Fig — Numbers indicate the mean number of mutations, and colour gradient indicates mean fitness after 1,000 (top) and 2,000 generations (bottom; initial fitness is 1). All mutations are deleterious, either with a fixed fitness effect (sd; left; n = 10 simulations per parameter combination) or with exponentially distributed effects with mean sd (right; n = 3 simulations per parameter combination). (TIF) [file pbio.3000617.s008.tif]

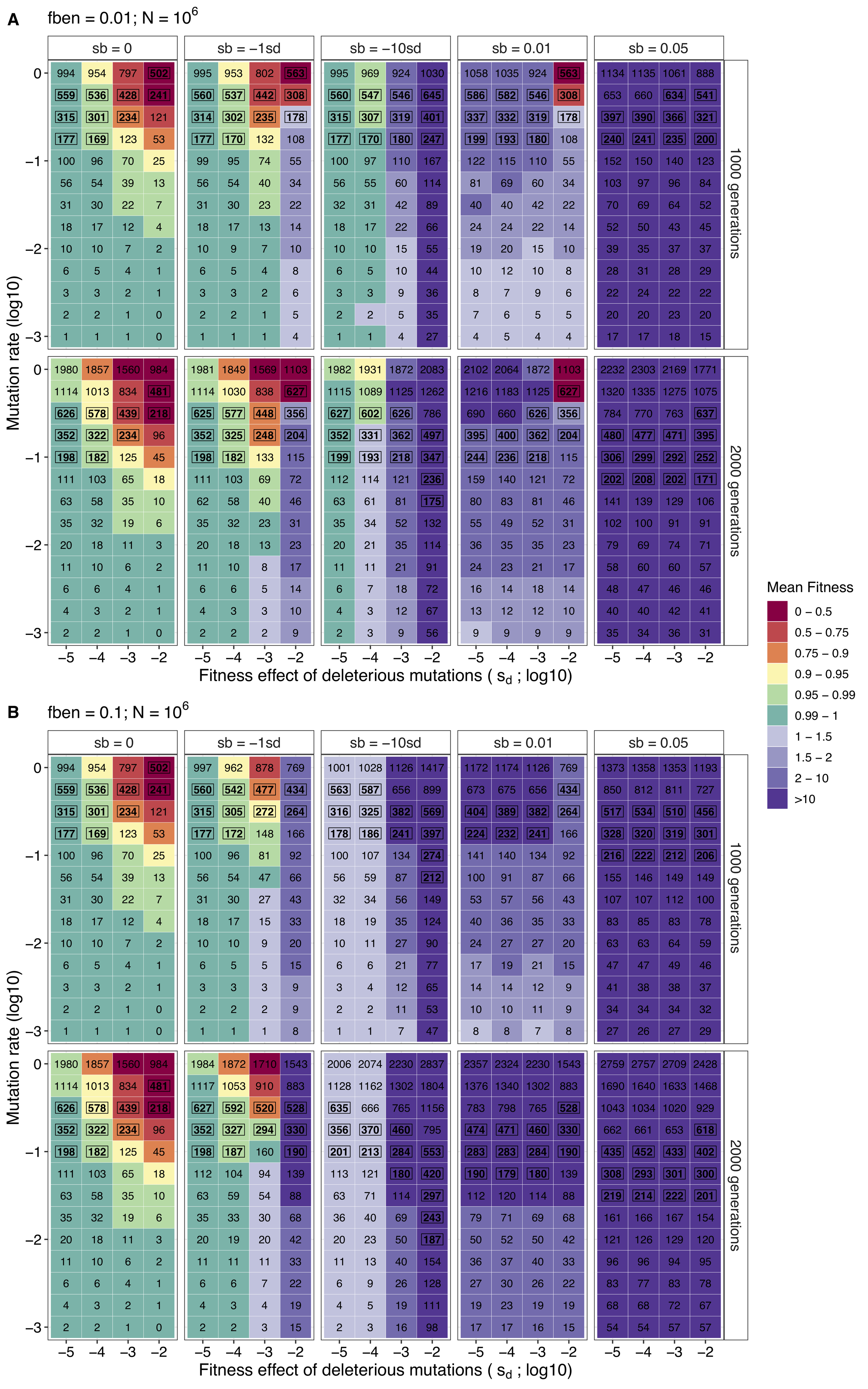

Supplement: S9 Fig — Either mutations were all deleterious (fben = 0) or a fraction (fben) of 1% (A) or 10% (B) was beneficial. The fitness effects of deleterious (sd) and beneficial (sb) mutations were drawn from two independent exponential distributions. Numbers indicate the mean number of mutations, and the colour gradient indicates mean fitness after 1,000 (top row) and 2,000 generations (bottom row; fitness starts at 1). Different sb values are represented in different columns (n = 3 simulations per parameter combination). See S5 Table for the mean and standard deviation of fitness and the number of mutations. (TIF) [file pbio.3000617.s009.tif]

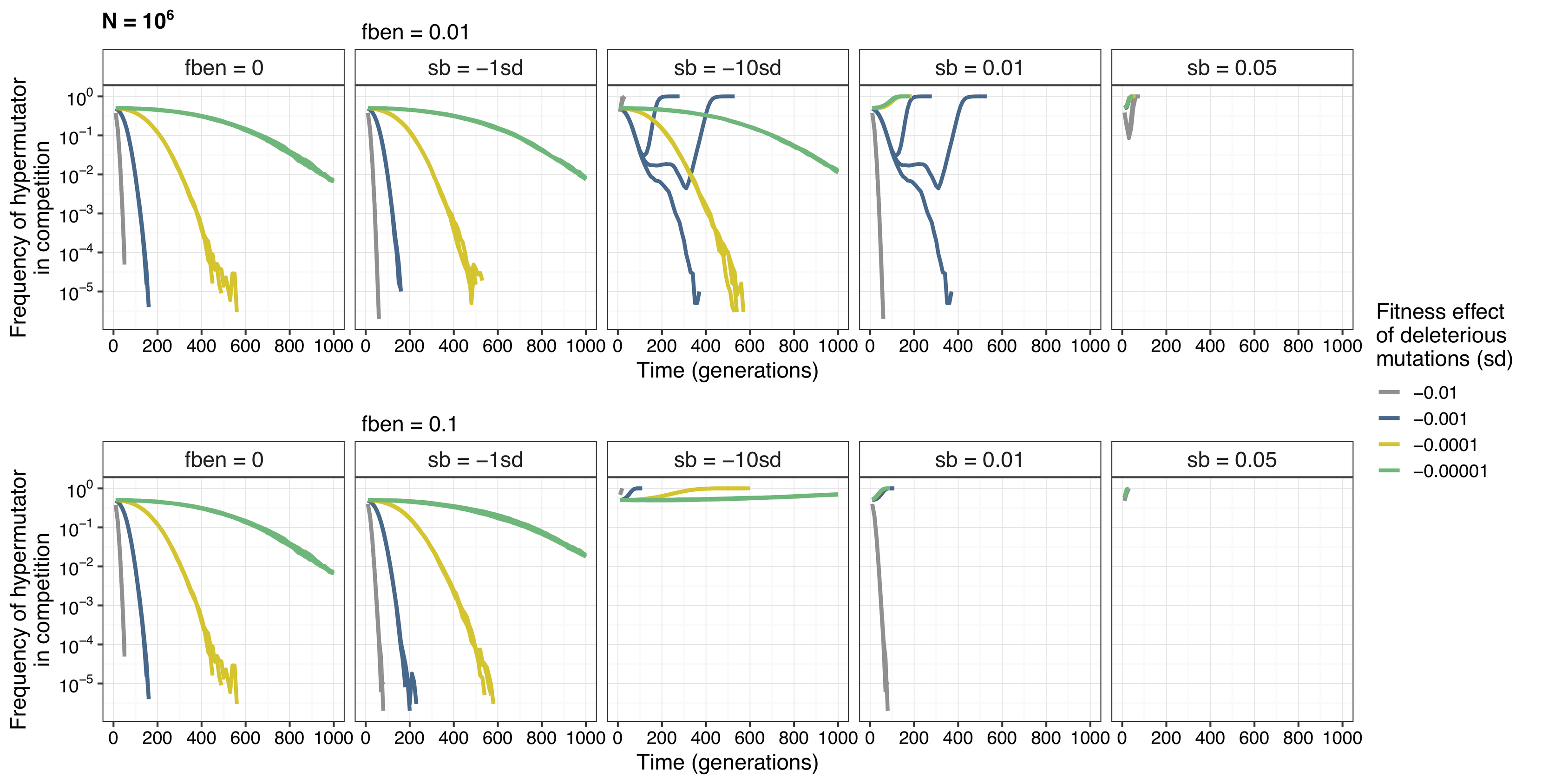

Supplement: S10 Fig — All populations had 106 individuals, and either mutations were all deleterious (left column) or a fraction (fben) of 1% (top) or 10% (bottom row) was beneficial. The fitness effects of deleterious (sd) and beneficial (sb) mutations were drawn from two independent exponential distributions. (TIF) [file pbio.3000617.s010.tif]

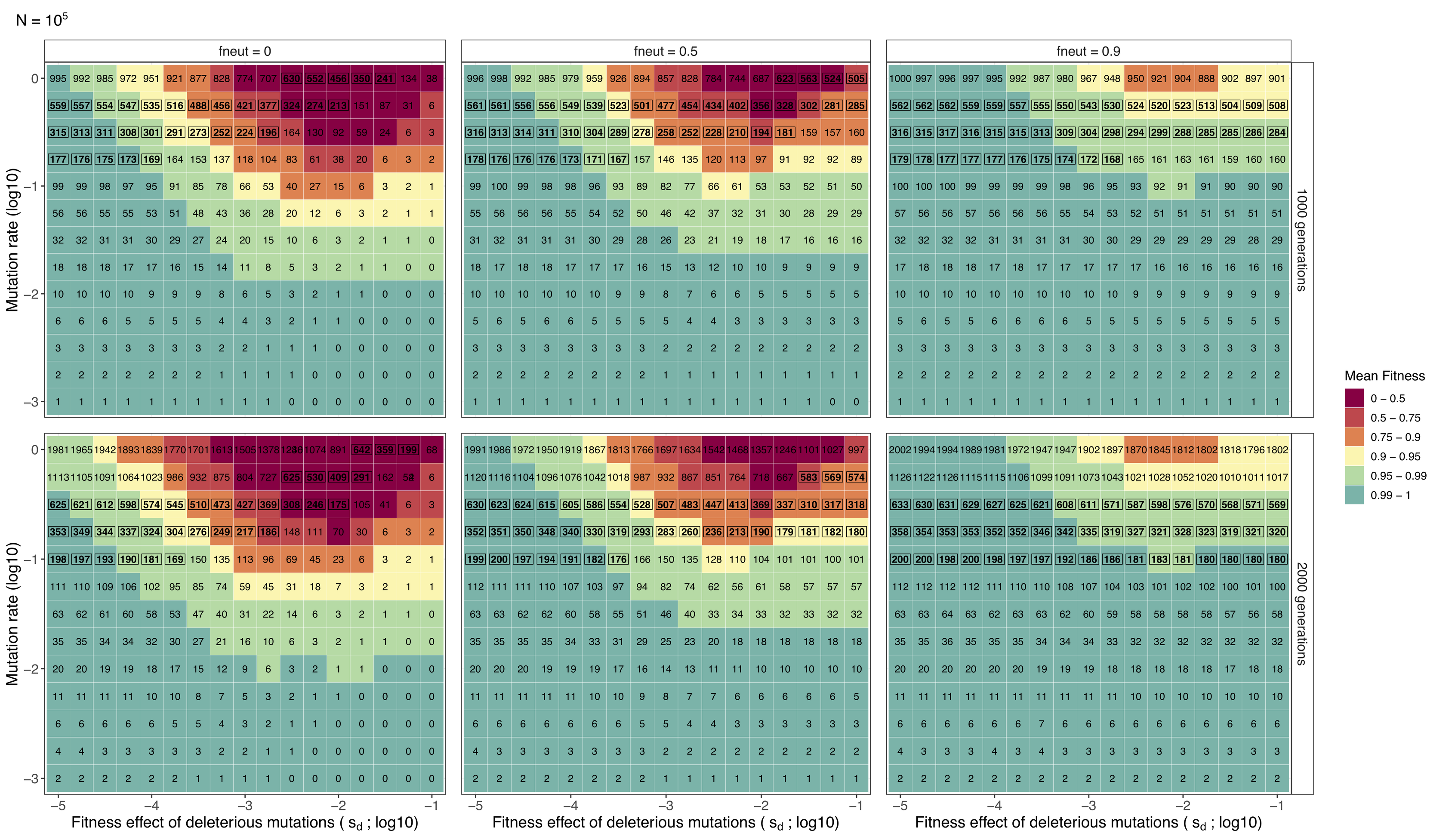

Supplement: S11 Fig — Numbers indicate the mean number of mutations, and colour gradient indicates mean fitness after 1,000 (top) and 2,000 generations (bottom; fitness starts at 1). Mutations were either all deleterious with a fixed fitness effect (left) or neutral, with the fraction of neutral mutations (fneut) being 50% or 90% (middle and right panels; n = 10 simulations per parameter combination when fneut = 0; n = 3 simulations per parameter combination when fneut = 0.5 or fneut = 0.9). See S8 Table for the mean and standard deviation of fitness and the number of mutations. (TIF) [file pbio.3000617.s011.tif]

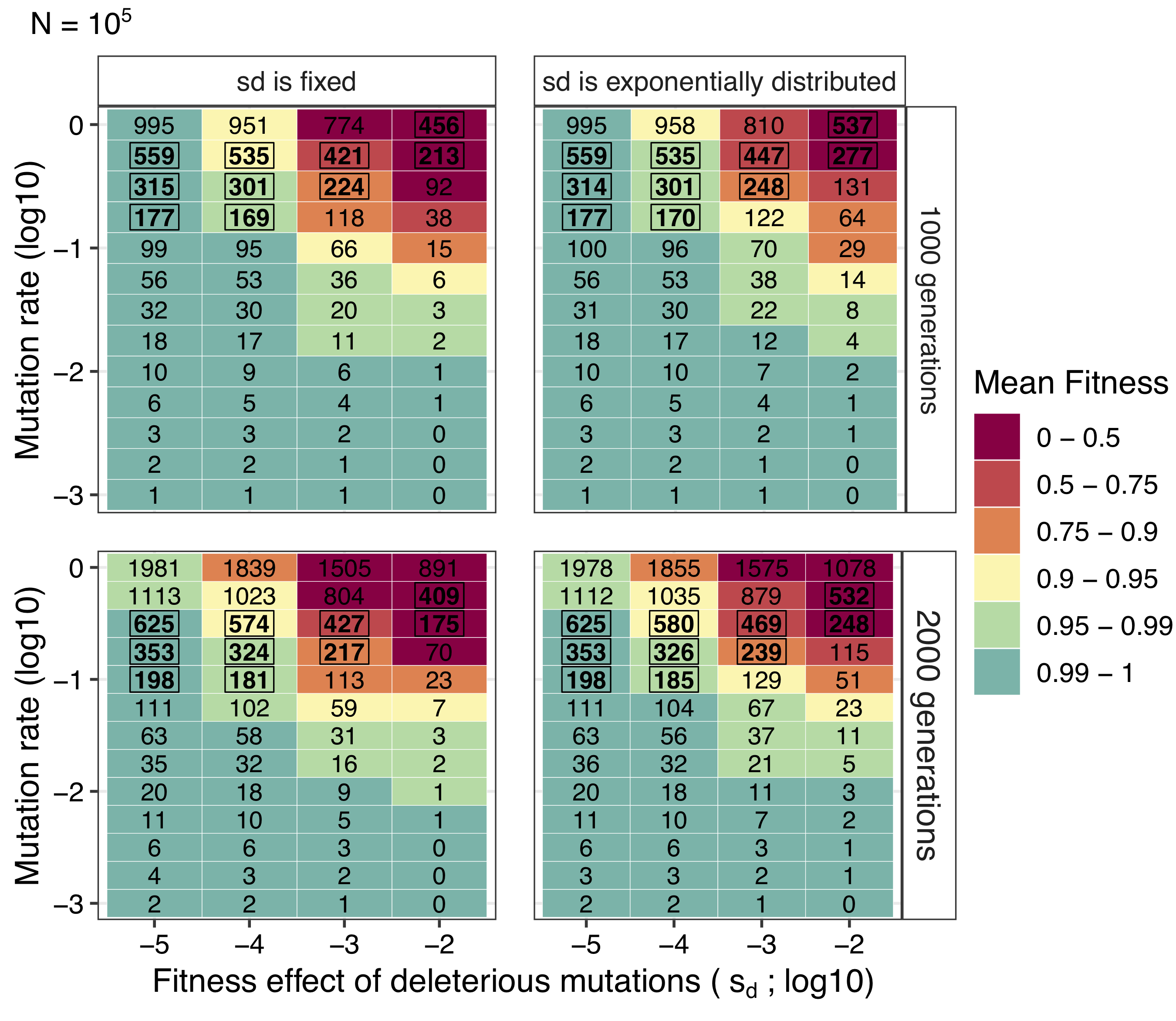

Supplement: S12 Fig — Numbers indicate the mean number of mutations, and colour gradient indicates mean fitness after 1,000 (top) and 2,000 generations (bottom; fitness starts at 1). All mutations are deleterious, either with a fixed fitness effect (sd; left; n = 10 simulations per parameter combination) or with exponentially distributed effects with mean sd (right; n = 3 simulations per parameter combination). (TIF) [file pbio.3000617.s012.tif]

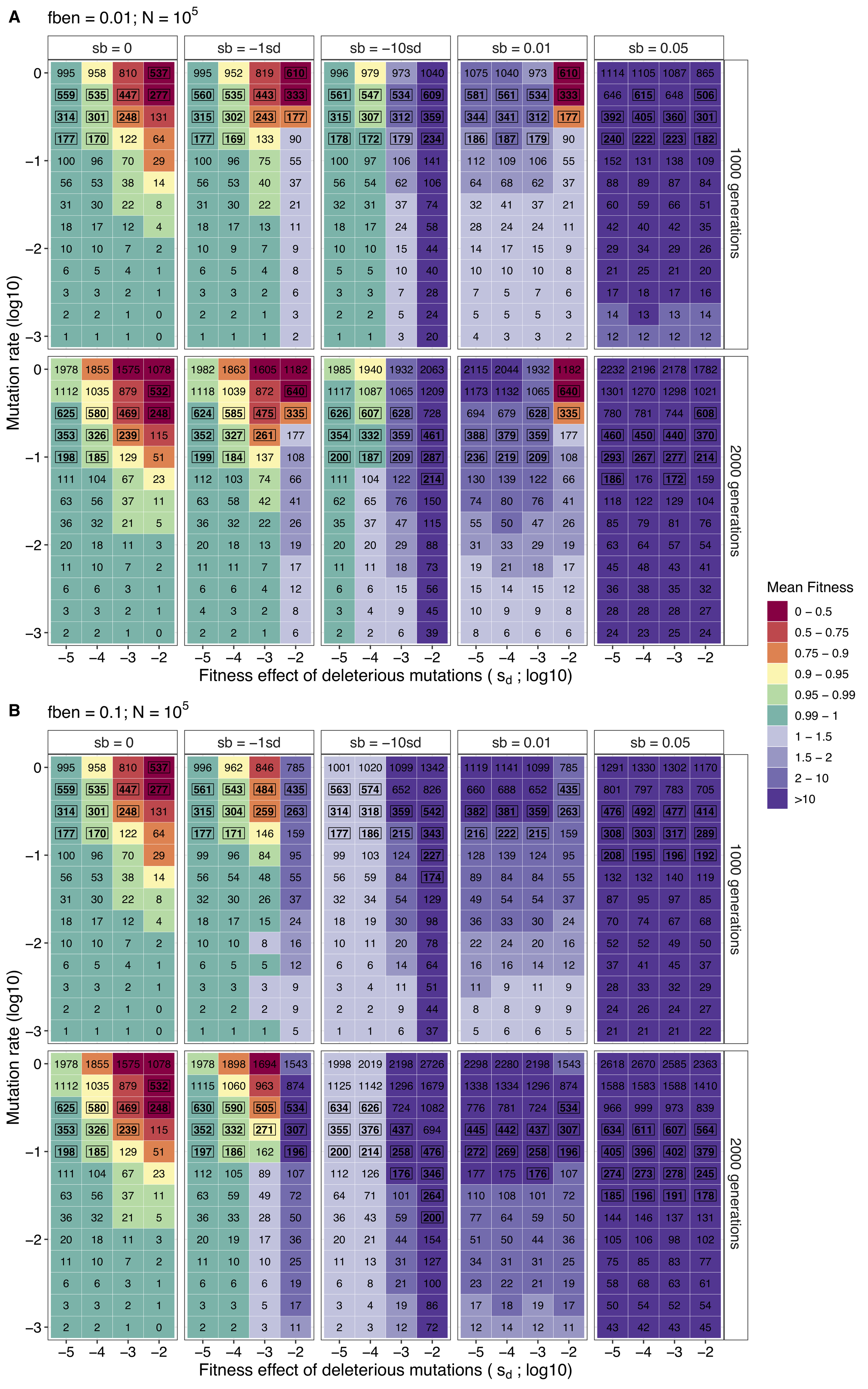

Supplement: S13 Fig — Either mutations were all deleterious (fben = 0) or a fraction (fben) of 1% (A) or 10% (B) was beneficial. The fitness effects of deleterious (sd) and beneficial (sb) mutations were drawn from two independent exponential distributions. Numbers indicate the mean number of mutations, and the colour gradient indicates mean fitness after 1,000 (top row) and 2,000 generations (bottom row; fitness starts at 1). Different sb values are represented in different columns (n = 3 simulations per parameter combination). See S9 Table for the mean and standard deviation of fitness and the number of mutations. (TIF) [file pbio.3000617.s013.tif]

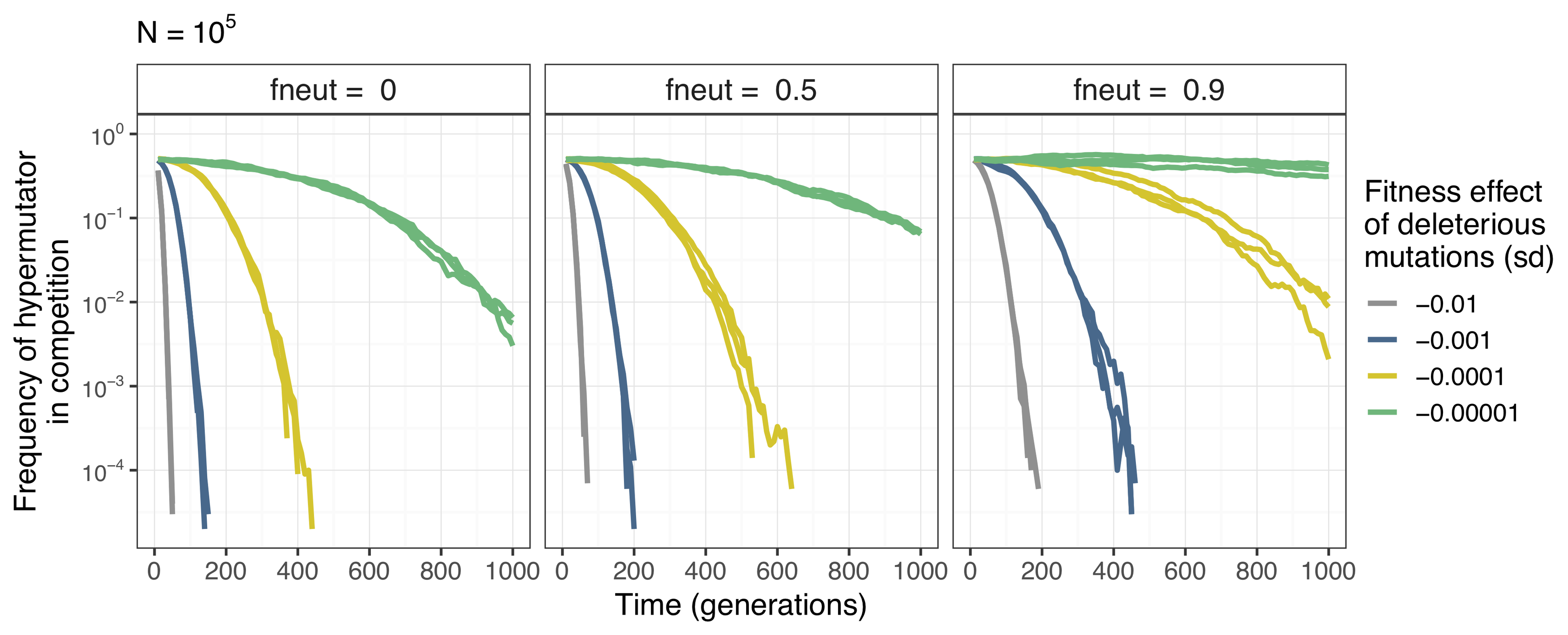

Supplement: S14 Fig — All populations had 105 individuals, and mutations were either all deleterious with a fixed fitness effect (left) or neutral, with the fraction of neutral mutations (fneut) being 50% or 90% (middle and right panels; n = 3 simulations per parameter combination). (TIF) [file pbio.3000617.s014.tif]

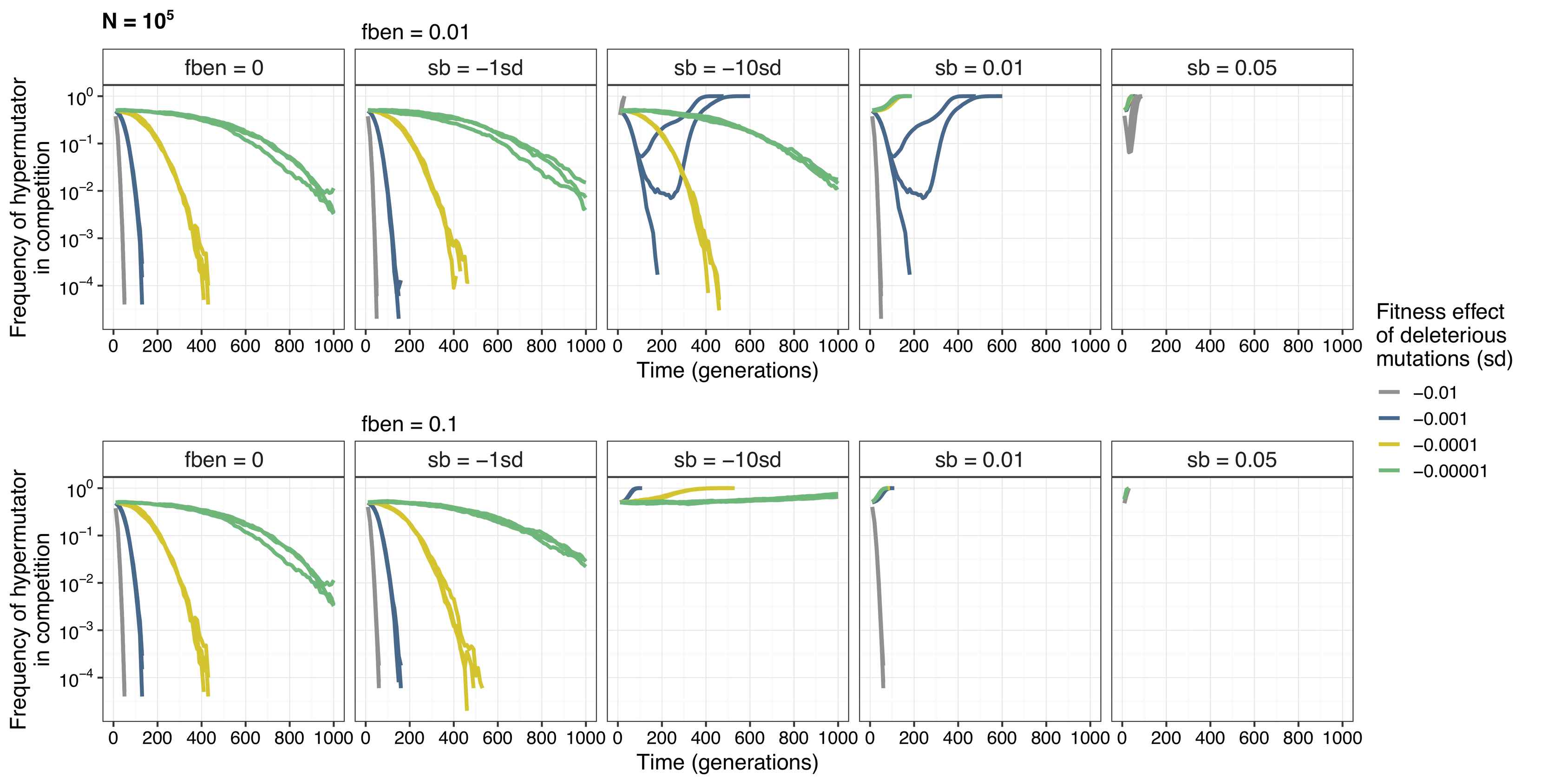

Supplement: S15 Fig — All populations had 105 individuals, and either mutations were all deleterious (left column) or a fraction (fben) of 1% (top) or 10% (bottom row) was beneficial. The fitness effects of deleterious (sd) and beneficial (sb) mutations were drawn from two independent exponential distributions. (TIF) [file pbio.3000617.s015.tif]

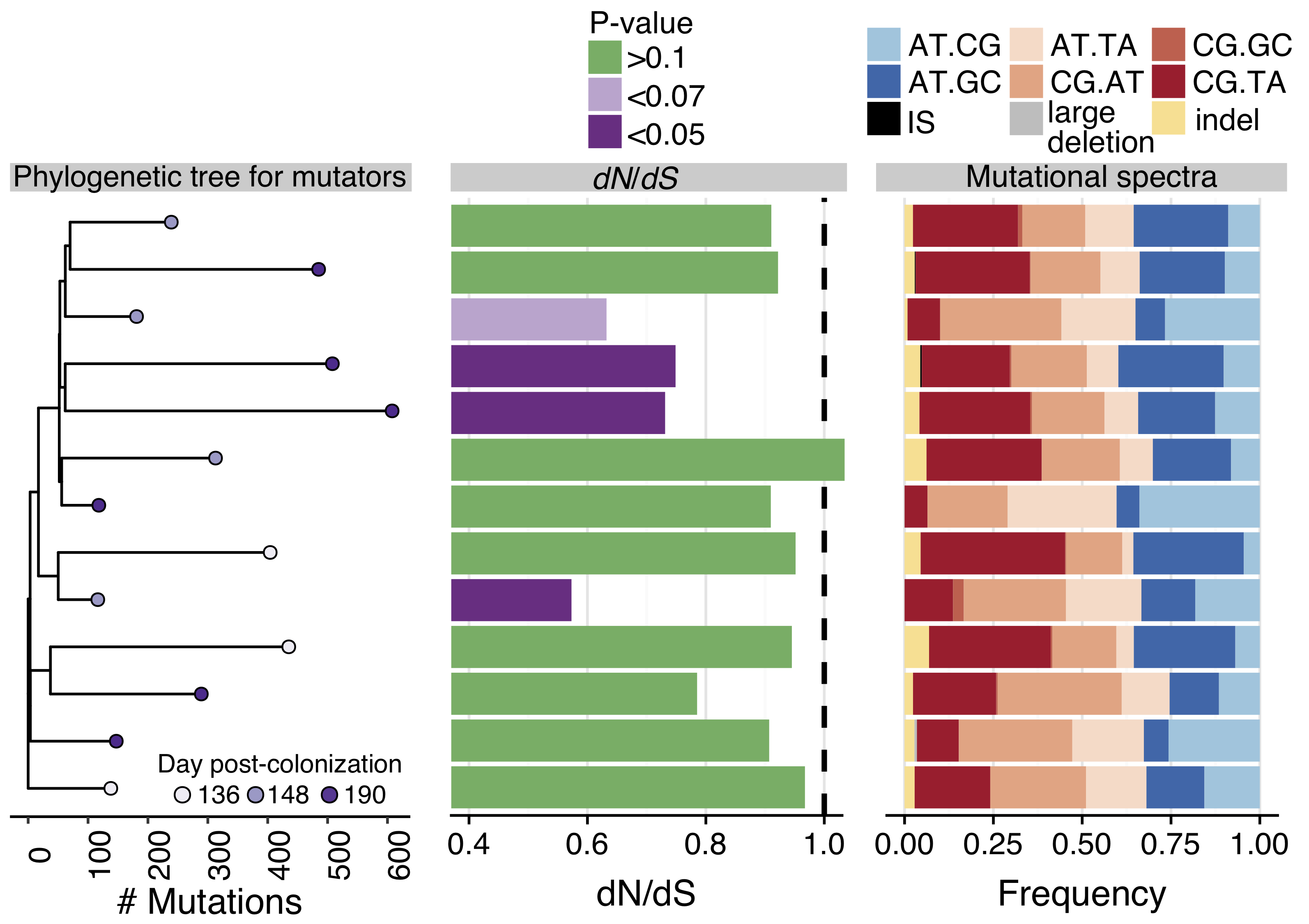

Supplement: S16 Fig — (A) Phylogenetic tree for the mutator clones sequenced from mouse 1. (B) dN/dS with colours highlighting whether this is significantly different from neutral expectation. (C) Mutational spectra. Mutations accumulated at the tips were chosen for this analysis as a way to avoid counting the same mutation multiple times. (TIF) [file pbio.3000617.s016.tif]

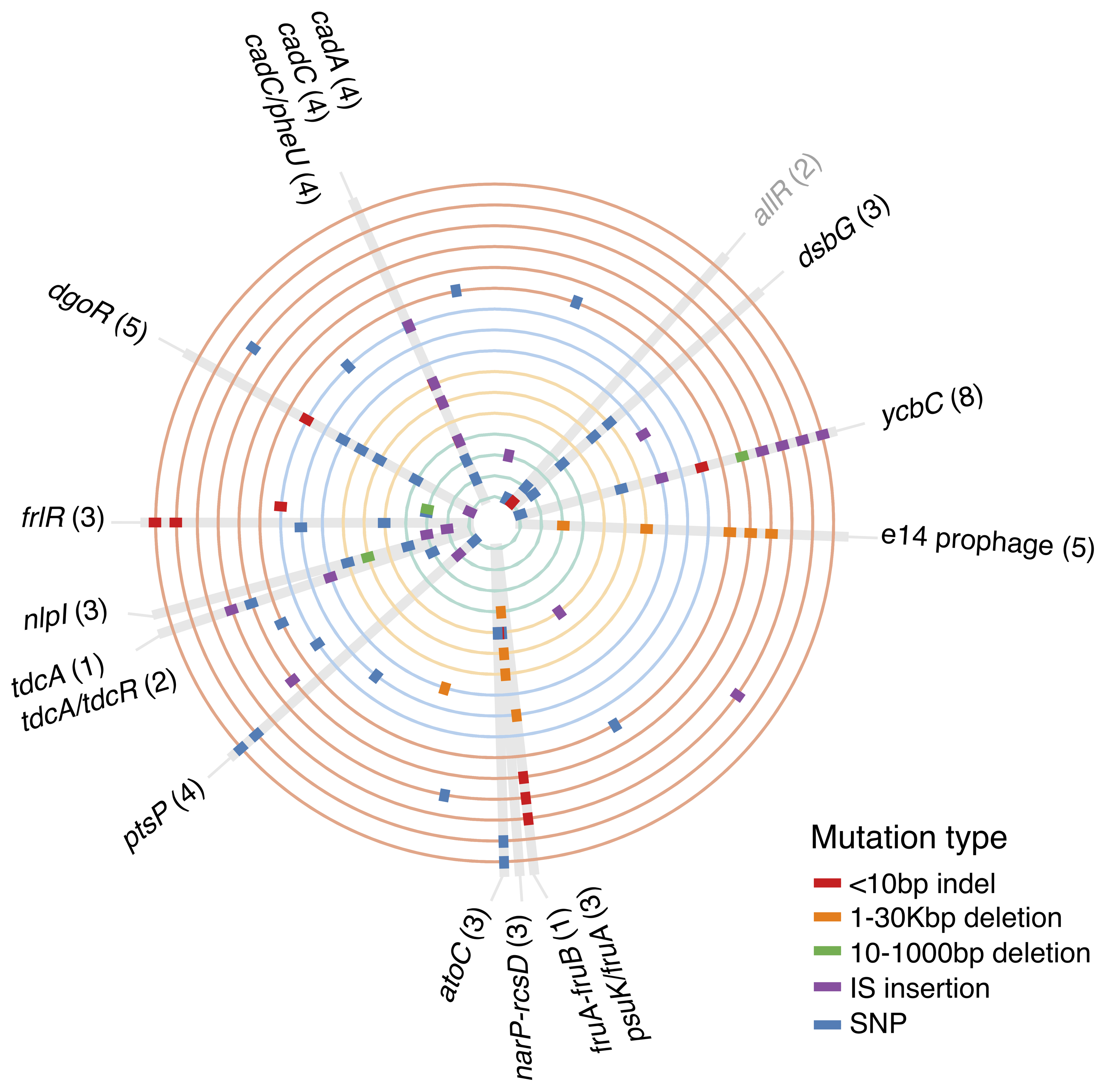

Supplement: S17 Fig — Circles represent genomes, different coloured circles represent different mice (orange, mouse 1; blue, mouse 2; yellow, mouse 3; green, mouse 4). Grey rectangles highlight mutations that were parallel across different mice (gene labels in black) or for which multiple alleles were found within the same mouse (gene labels in grey). Small rectangles crossing a particular circle indicate mutations, with colours representing different mutation types. Note that for the figures in the main text, we always show 5 nonmutator clones from mouse 1, and there are 6 in this figure. This is because we sequenced one clone at day 190 from the YFP background. As this is not genetically related to the ancestral from which the mutators emerged, we do not show this clone in the main figures. YFP, yellow fluorescent protein. (TIF) [file pbio.3000617.s017.tif]

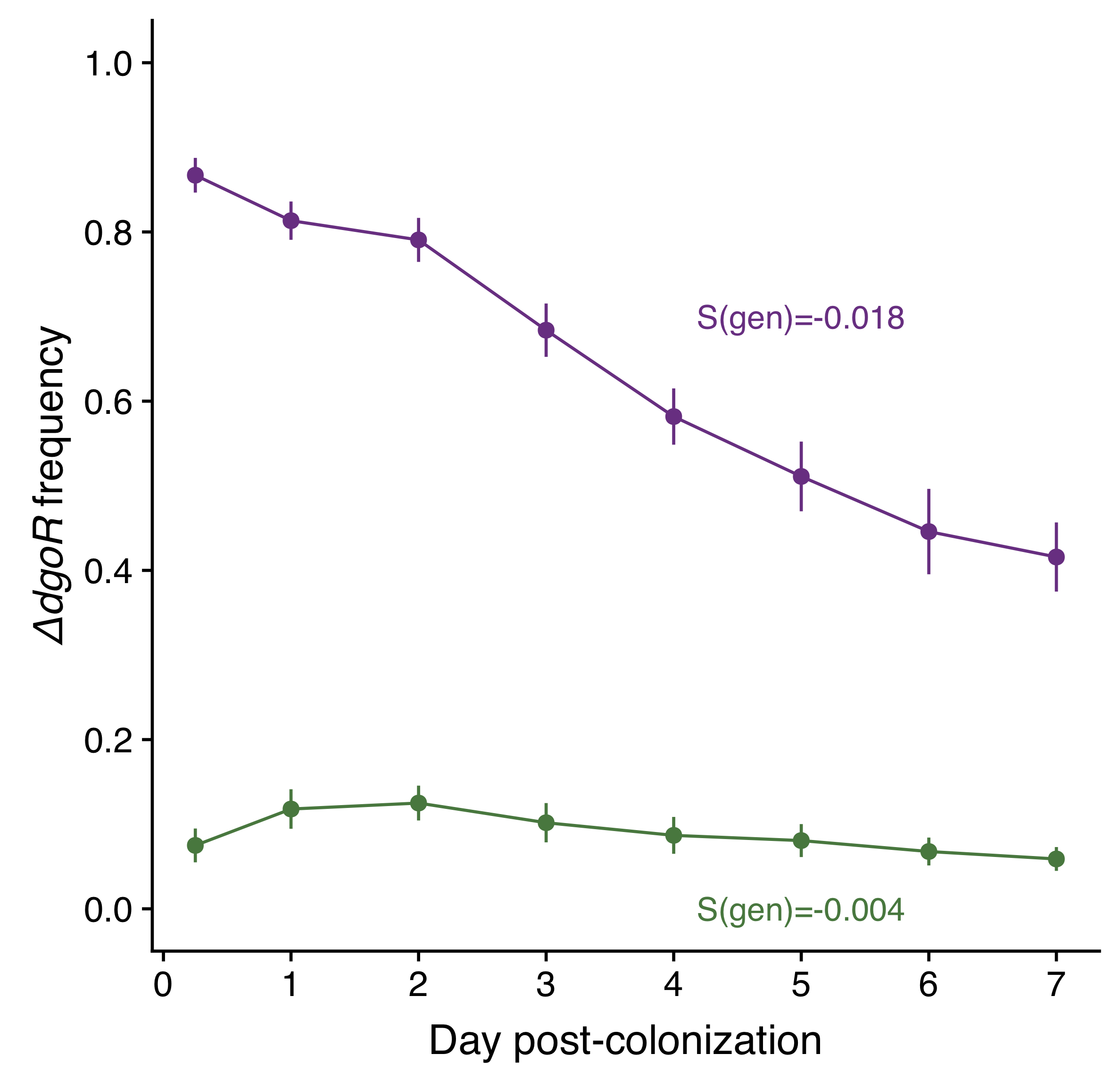

Supplement: S18 Fig — The selective coefficient per generation (S(gen)) is indicated above each line. (TIF) [file pbio.3000617.s018.tif]
